# Macrophages orchestrate tail regeneration in lizards *Scincella tsinlingensis* through spatiotemporal control of immunity, oxidative stress, and myogenesis

**DOI:** 10.3389/fcell.2026.1873713

**Published:** 2026-08-06

**Authors:** Qing Li, Yiwen Zhang, Tingting Shi, Rongnan Li, Yongqing Yang, Chun Yang

**Affiliations:** School of Life Sciences, Shanxi Normal University, Taiyuan, China

**Keywords:** macrophages, matrix metalloproteinases, regeneration, *Scincella tsinlingensis*, transcriptome

## Abstract

**Background:**

Macrophages facilitate blastema and wound epithelium formation by clearing debris and remodeling the immune and extracellular matrix. The M1-to-M2 transition is well documented in amphibians, but its occurrence and timing in diverse lizards remain unclear. This study examined macrophage function during tail regeneration in *Scincella tsinlingensis*.

**Methods:**

A tail amputation model was established in *S. tsinlingensis*, and macrophages were depleted using clodronate liposomes (L-Clodronate). Transcriptomic analysis was performed during the wound epithelialization phase, followed by qRT-PCR and immunohistochemistry to assess immune responses, chemokine signaling, oxidative balance, myogenic regulation, and matrix metalloproteinase (MMP) activity. MMP function was further examined using GM6001 inhibition.

**Results:**

Macrophage depletion severely impaired tail regeneration. By 21 days post-amputation (dpa), control regenerates reached 6.1 ± 2.2 mm, whereas depleted tails grew only 0.5 ± 0.1 mm (P < 0.0001). Depletion delayed wound epithelialization and prevented blastema formation. Macrophage recruitment peaked later in depleted animals (86.8 ± 13.8 at 7 dpa) than in controls (148.4 ± 7.1 at 3 dpa). CTSK positive osteoclasts were reduced by ∼47% (53 ± 5.6 vs. 100.3 ± 18.1, P < 0.05). Transcriptomic analysis identified 341 DEGs, with downregulated genes enriched in type I interferon, desmosome, and TLR/NOD pathways. *TLR4* was a hub gene. qPCR and immunohistochemistry confirmed disrupted M1-to-M2 transition, suppressed cytokines (TNFα, IL-6, IL-10, TGFβ1), dysregulated CXCL12/CXCR4, oxidative imbalance (gp91phox), failed myogenesis (decreased MYOD1, persistent PAX7), and reduced Ctsk, MMP2, MMP9. GM6001 matrix metalloproteinase inhibition delayed regeneration (10.2 ± 1.2 vs. 1.5 ± 0.1 mm at 28 dpa, P < 0.0001) with compensatory upregulation of MMP2/3/9.

**Conclusion:**

Macrophages serve as central timing regulators of tail regeneration in *S. tsinlingensis*. Their depletion disrupts the early inflammatory switch, impairs blastema formation, and severely limits outgrowth within the 21-day observation period, whereas MMP inhibition only slows regeneration. Macrophages coordinate the immune, myogenic, and matrix remodeling programs essential for successful tissue restoration, highlighting their non-redundant role in reptile regeneration.

## Introduction

Lizards and mammals share advanced immune systems and similar inflammatory responses to injury, so lizards are useful models for appendage regeneration in a complex wound environment (Londono et al., 2020). Among adult tetrapods, lizards are unique in having both a regenerating tail and a non-regenerating limb in the same individual (Lozito and Tuan, 2017; Alibardi, 2018), a contrast that could be used to compare the cellular and molecular mechanisms that suppress scarring, promote blastema formation, and enable successful regrowth (Lozito et al., 2025).

Work in amphibians has shown that macrophages, especially the pro-regenerative M2 subtype, are required for regeneration (Godwin et al., 2013; Godwin and Rosenthal, 2014). Similar macrophage-dependent processes support wound healing in mammals (Mescher, 2017; Krzyszczyk et al., 2018). In the regenerating tail blastema of the lizard *Podarcis muralis*, macrophages are abundant among mesenchymal cells; some are phagocytic while others are not, suggesting additional roles beyond debris removal. The presence of M2-like macrophages supports the idea that the regenerative blastema creates an immuno-privileged niche that allows controlled growth without tumor risk (Alibardi, 2020). In zebrafish, peripheral macrophages also fine-tune inflammation to promote regeneration (Morales and Allende, 2019). Macrophage polarization into distinct M1 and M2 phenotypes is a key determinant of repair versus fibrosis (Murray, 2017), and type 2 immunity, including M2 macrophages, plays central roles in tissue repair and fibrosis (Gieseck et al., 2018). Persistent activation of macrophages and myofibroblasts, together with mechanical signals, drives fibrotic outcomes (Pakshir and Hinz, 2018).

Research on more than 30 lizard species from six families—including *Anolis carolinensis* (Hutchins et al., 2014; Hutchins et al., 2016; Palade et al., 2018), *Eublepharis macularius* (Payne et al., 2017; Gilbert and Vickaryous, 2018), and *Gekko japonicus* (Liu et al., 2015)—has defined the histochemical, biochemical and cellular features of tail regeneration and begun to uncover its molecular basis (Alibardi, 2014). Whole-genome sequences for these species (Alföldi et al., 2011; Liu et al., 2015; Xiong et al., 2016) and for *Shinisaurus crocodilurus* (Gao et al., 2017) provide essential genomic resources. Multi-omics and single-cell analyses have further identified key signaling molecules and pathways active during regeneration (Londono et al., 2020; Vonk et al., 2023). However, *Scincella* lizards—particularly the ovoviviparous Chinese endemic *Scincella tsinlingensis*—remain understudied. This species offers a valuable model for epimorphic regeneration under ovoviviparous conditions. Recent work showed that its tail regeneration follows a structural pattern conserved among lizards (Yang et al., 2022; Wang et al., 2026).

Successful tissue regeneration depends on extracellular matrix (ECM) remodeling, which requires coordinated expression of matrix components and matrix metalloproteinases (MMPs) (Fingleton, 2017). Basal epithelial cells at the wound site secrete MMPs, including acid hydrolases and proteases, that degrade ECM components such as collagen and laminin. This activity likely facilitates cell migration and intercellular communication by modifying the basement membrane (Zhao et al., 2022). Londono et al. showed that pharmacological inhibition of MMPs severely impairs tail regeneration in lizards, and single-cell RNA-seq comparison of lizard and mouse immune cells highlighted macrophages and macrophage-derived MMPs as key regulators (Londono et al., 2020). In *G. japonicus*, tail amputation induces strong upregulation of macrophage migration inhibitory factor (MIF) at the injury site, together with increased MMP1 and MMP3 expression in astrocytes; MIF appears to promote astrocyte migration via MMP1/3, facilitating outgrowth (Zhang et al., 2023). Khaire et al. (2021) showed that COX-2 inhibition alters *Mmp* and *Fgf* gene expression during tail regeneration, with a positive correlation between COX-2 and gelatinase activities, pointing to a critical interplay among COX-2, MMPs and FGF signaling during initiation of regeneration.

As key effectors of innate immunity, macrophages play dual roles in tissue repair: they clear pathogenic and apoptotic debris by phagocytosis and shape the regenerative niche through secretion of immune mediators that regulate inflammation and facilitate ECM remodeling. Tissue-resident macrophages contribute to homeostasis and repair (Davies et al., 2013), and their timely activation is critical for wound healing (Eming et al., 2014). In mammals, macrophages are required for digit tip regeneration (Simkin et al., 2017), and they regulate muscle growth and regeneration (Tidball, 2017). Reactive oxygen species generated by NADPH oxidases, including gp91phox, modulate inflammatory and repair processes (Bedard and Krause, 2007; Hecker et al., 2009), and the transcription factor Nrf2 controls antioxidant responses (Tonelli et al., 2018). ROS also promote angiogenesis via NADPH oxidase (Ushio-Fukai and Nakamura, 2008). Successful limb regeneration in urodeles involves coordinated inflammatory and ECM signals (Stocum, 2017). Although macrophage polarization into M1/M2 phenotypes is essential for amphibian regeneration, its spatiotemporal regulation in lizard models remains poorly characterized. Previous studies in *S. tsinlingensis* described dynamic changes in immune cells and factors during tail regeneration (Yang et al., 2024), but an integrated analysis focusing on macrophages is lacking. In the present study, we used a tail regeneration model with macrophage depletion via intraperitoneal L-Clodronate to ask how M1/M2 phenotypic switching regulates transcriptional networks across regenerative stages, especially the inflammatory and proliferative phases. We also examined the consequences of macrophage depletion on chemokine gradients, redox homeostasis and muscle satellite cell activity, and explored whether compensatory mechanisms exist in response to macrophage loss and whether they resemble mammalian repair pathways.

## Materials and methods

### Lizards

Adult *S. tsinlingensis* (snout-vent length 4.8–5.7 cm) were collected from Qiliyu forest in Taiyue Mountain, Shanxi, China (36°21′-36°45′N, 110°40′-112°21′E). All experiments followed the guidelines of the National Institutes of Health Guide for the Care and Use of Laboratory Animals (eighth ed, National Research Council, 2011) and were approved by the Animal Ethics Committee of Shanxi Normal University (SXNU-STEC-2024–0316). Animal husbandry was as described previously (Yang et al., 2022; Yang et al., 2024). Briefly, adult lizards were kept in soil-filled terrariums at 40%–60% humidity, under a photoperiod of 10 h light at 24 °C and 14 h dark at 19 °C. Tap water and *Tenebrio molitor* larvae were provided daily. Tails were autotomized 2 cm from the cloaca. Regenerated tails were collected at different times by inducing autotomy just proximal to the regenerated portion.

### *In vivo* macrophage depletion

**FIGURE 1:**
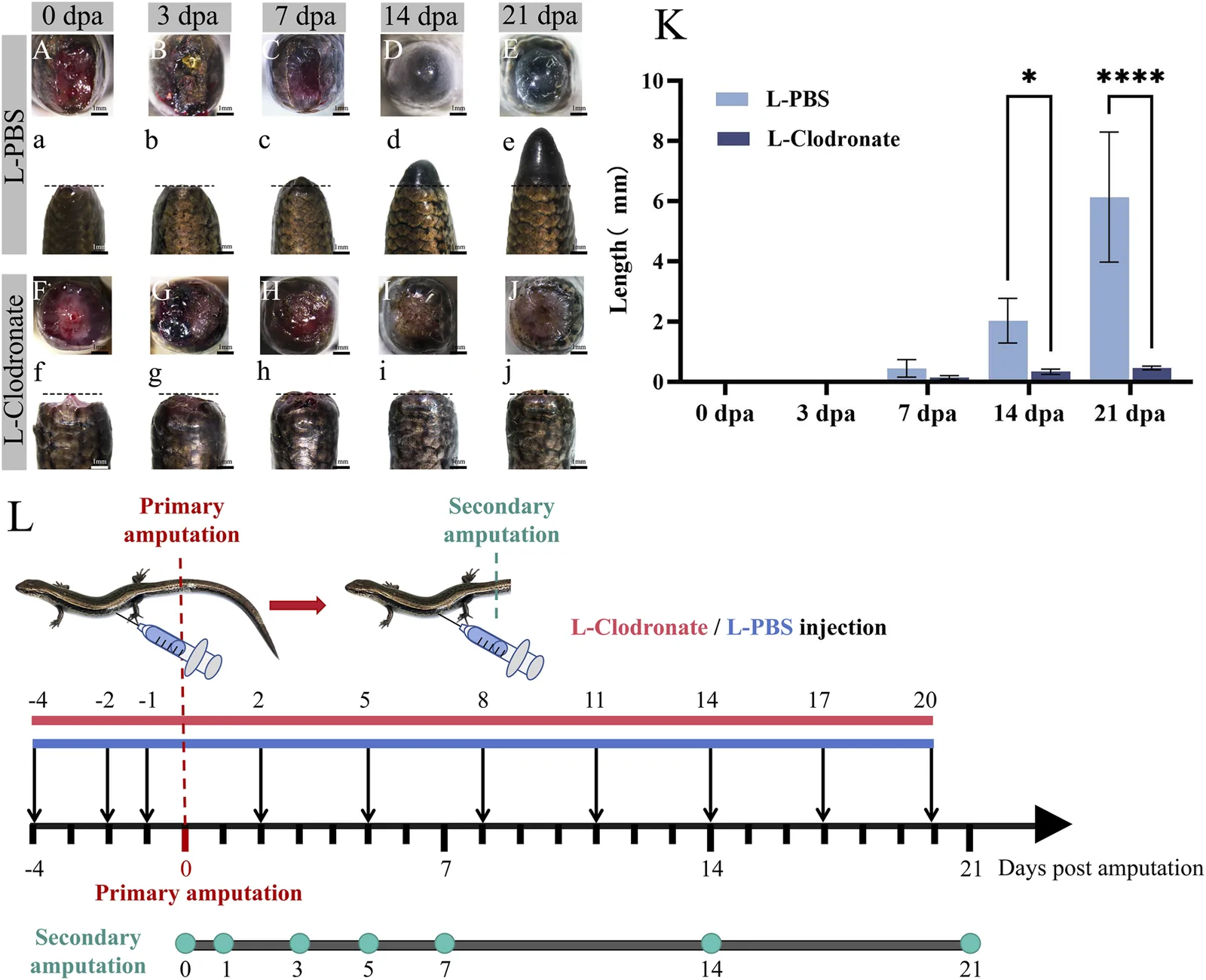
Tail regeneration in *S. tsinlingensis* is impaired upon macrophage depletion. **(A–J)** External morphology of regenerating tails after clodronate-mediated macrophage ablation. **(K)** Quantification of regenerated tail length. **(L)** Experimental timeline and groups: liposome injections (L-PBS vs. L-Clodronate, both labeled with L-Dil) were administered pre-amputation (4, 2, 1 day) and continued every 2 days thereafter.

We followed a previous protocol for systemic phagocyte depletion during tail regeneration (Londono et al., 2020). Lizards received intraperitoneal (IP) injections of L-α-phosphatidylcholine/cholesterol liposomes containing clodronate (0.125 mg/g) at 4 days, 2 days and 1 day before tail amputation, and then every 3 days thereafter. Control animals received liposomes containing PBS. To fluorescently label phagocytic cells, lizards were IP injected with L-α-phosphatidylcholine/cholesterol/DiI liposomes (Liposoma, NL) together with each treatment injection. Secondary tail amputations were performed at 0, 1, 3, 5, 7, 14 and 21 days post-amputation (dpa). Six regenerated tails were collected per time point. The injection scheme and sampling timeline are shown in Figures 1A–L.

### MMP inhibition

The broad-spectrum MMP inhibitor GM6001 (InvivoChem, United States of America) was delivered by IP injection (100 mg/kg) together with L-Dil every other day for 4 weeks. Control lizards received DMSO and L-Dil. After 4 weeks, regenerated tail lengths were photographed and measured in the GM6001 and solvent control groups. Regenerated tail tissues harvested at 7 dpa were embedded and sectioned.

### Histology and immunohistochemistry

Regenerated tail tissues from macrophage-depleted animals were fixed in 4% paraformaldehyde at 4 °C for 3 days, decalcified (Leica Surgipath Decalcifier I) with constant shaking for 3 days, dehydrated in graded ethanol, cleared in xylene (3 times, 1 h each) and embedded in paraffin wax. Serial sections (10 µm) were placed on polylysine slides and used for hematoxylin-eosin (H&E) staining.

To evaluate the impact of macrophage depletion, we examined by immunohistochemistry the expression of key markers for immune cells, cytokines, chemokines, oxidative stress and fibroblasts. Deparaffinized and rehydrated sections underwent antigen retrieval and blocking, followed by incubation with primary antibodies against CD86 (Cloud-clone, PAA824, 1:100), iNOS (Cloud-clone, PAA837, 1:100), CD206 (Boster, A02285-2, 1:100), CD163 (Beyotime, AF6453, 1:100), TNFα (Beyotime, AF8208, 1:100), IL6 (Beyotime, AF7236, 1:100), IL10 (Beyotime, AF7248, 1:100), TGFβ (Beyotime, AF8145, 1:100), CCL4 (Cloud-clone, PAA093Mu01, 1:100), CXCL12 (Beyotime, AF6612, 1:100), CXCR4 (Beyotime, AF6621, 1:100), gp91phox (Beyotime, AF7596, 1:100), MYOD1 (Beyotime, AF7539, 1:100) and PAX7 (Beyotime, AF2638, 1:100) at 4 °C for 24 h. After warming to room temperature and washing, sections were treated with species-specific secondary antibodies at 37 °C for 4 h. Signals were developed using a biotinylated secondary antibody and SABC solution (37 °C, 30 min), followed by diaminobenzidine substrate reaction. Images were acquired with a microscope.

For immunofluorescence, we examined markers of macrophages and MMPs: CTSK (Cloud-clone, PAA267Mu01, 1:100), MMP2 (Boster, A02285-2, 1:100), MMP3 (Cloud-clone, PAA101Mu01, 1:100) and MMP9 (Cloud-clone, PAA553Rb51, 1:100). Paraffin sections were deparaffinized, rehydrated, and delineated with a hydrophobic barrier. Antigen retrieval was performed with a composite digest solution for 20 min. Sections were permeabilized with 0.5% Triton X-100 for 20 min, blocked with 5% BSA, and incubated with primary antibodies at 4 °C for 24 h. After washing, corresponding fluorescently conjugated secondary antibodies (SABC-DyLight Cy3, SA1074, SA1072; SABC-FITC (POD), SA1082, SA1080; Boster) were applied at 37 °C for 1 h in the dark. Nuclei were counterstained with DAPI and sections were mounted for immediate imaging.

### RNA extraction, library preparation, and sequencing

We chose 7 dpa based on our lab’s previous work on tail regeneration in *S. tsinlingensis*. At this stage, the animal is transitioning from the late inflammatory phase to early cell proliferation, preparing for blastema formation (Yang et al., 2022). Given macrophages’ broad roles in inflammation, proliferation, angiogenesis, and cartilage formation, we felt this time point provides a useful starting snapshot. We extracted total RNA from tails of the 0 dpa, 7 dpa L-Clodronate, and 7 dpa L-PBS groups with the miRNeasy Mini Kit (Qiagen). RNA integrity and concentration were checked on an Agilent Bioanalyzer 2100 (RNA Nano 6000 Assay Kit) and a Qubit 2.0 Fluorometer (Qubit RNA Assay Kit). After quality control, three RNA samples per group were sent to Biomarker Technologies Corporation (Beijing, China) for library construction and sequencing. mRNA was purified and fragmented; double-stranded cDNA was then synthesized, end-repaired, adenylated, and ligated to NEBNext adapters. Libraries were purified with the AMPure XP system, PCR-amplified, and cleaned up again. Library quality was verified on an Agilent Bioanalyzer 2100, and sequencing was done on an Illumina HiSeq 2000 platform.

### Transcriptome assembly, quantification, and annotation

Raw sequences were processed on the BMKCloud online platform (www.biocloud.net). We assembled clean reads *de novo* with Trinity (v2.5.1) to generate contigs. To reduce redundancy, we ran CD-HIT and TGICL on the contigs, clustering sequences with >90% similarity, and kept the longest transcript from each cluster as a unigene. Transcript abundance was calculated as FPKM using RSEM (v1.3.3). For annotation, we used DIAMOND against NR, KOG/COG/eggnog, Swiss-Prot, and KEGG. KEGG orthology analysis was done with KOBAS, GO annotation with InterProScan, and Pfam alignment with HMMER.

### Differential expression and enrichment analysis

We performed differential expression analysis with the EBSeq R package, using qvalue < 0.05 and |log2 fold change| > 1 as thresholds. GO enrichment analysis of DEGs was done with the topGO R package (Kolmogorov-Smirnov test). KEGG pathway enrichment was analyzed with KOBAS.

### Protein-protein interaction network

The STRING database (http://www.string-db.org) was used to predict protein-protein interactions (PPIs). Networks were visualized and analyzed with Cytoscape.

### qRT-PCR

Seven target mRNAs were randomly selected for qRT-PCR to validate the RNA-seq data. Primer sequences are listed in Supplementary Table S1. *Gapdh* served as the positive control. RNA extraction and quality assessment (A_260_/A_280_) were performed as above. Reverse transcription used the Aidlab TUREscript first Strand cDNA Synthesis Kit. qPCR was run on an analytikjena-qTOWER2.2 with a 10 µL reaction system, pre-denaturation (95 °C, 3 min) and 39 cycles of 95 °C for 10 s and 60 °C for 30 s. Expression ratios (2^-ΔΔCt^) from qRT-PCR were compared with FPKM values from RNA-seq to check consistency.

### Quantification and statistical analysis

Regenerated tail morphology was examined and photographed under a stereomicroscope. IHC and HE sections were imaged on a BX-40 optical microscope; IF sections on a BX-53 fluorescence microscope. Fluorescence images were processed with ImageJ and assembled with Adobe Photoshop. Positive cells for immune cells, cytokines, chemokines and oxidative stress markers were counted from three random 40× fields per section. Data were analysed in GraphPad Prism 9.5. IHC data were compared by two-way ANOVA followed by *post hoc* tests. For fluorescence quantification, three random 20× fields were analysed; mean fluorescence intensity was measured after uniform brightness/contrast adjustment in ImageJ, and statistical significance was determined by two-way ANOVA with *post hoc* test. qRT-PCR data were analysed by one-way ANOVA. P < 0.05 was considered significant. All values are shown as mean ± SD.

## Results

### Macrophage depletion severely impairs tail regeneration in *S. tsinlingensis*

At 0 dpa, both control and L-Clodronate groups looked similar: the wound exposed vertebrae, spinal cord, adipose tissue and muscle (Figures 1A,F). By 3 dpa, wound contraction had occurred and a coagulum covered the wound in both groups (Figures 1B,G). At 7 dpa, controls had lost the coagulum, developed a thin wound epithelium with visible blood vessels, and showed early signs of blastema formation (Figure 1C). In the depletion group, however, the coagulum remained, bleeding persisted, and proper epithelialization did not occur (Figure 1H). By 14 dpa, the control epithelium had become thicker, with clear pigmentation and blastema growth (Figures 1D, d). The depletion group only had a thin epithelium under the leftover coagulum, and the surface stayed flat with no signs of outgrowth (Figures 1I, i). At 21 dpa, controls showed clear elongation and skin shedding (Figures 1E, e). In contrast, the depletion group still had a thin, unpigmented epithelium and no blastema, resulting in a flat appearance with no obvious regrowth at this time point (Figures 1J, j). Meanwhile, control regenerates had grown to 6.1 ± 2.2 mm by 21 dpa, while the depletion group reached only 0.5 ± 0.1 mm (P < 0.0001; Figure 1K).

### Macrophage depletion impairs wound epithelialization and prevents blastema formation

**FIGURE 2:**
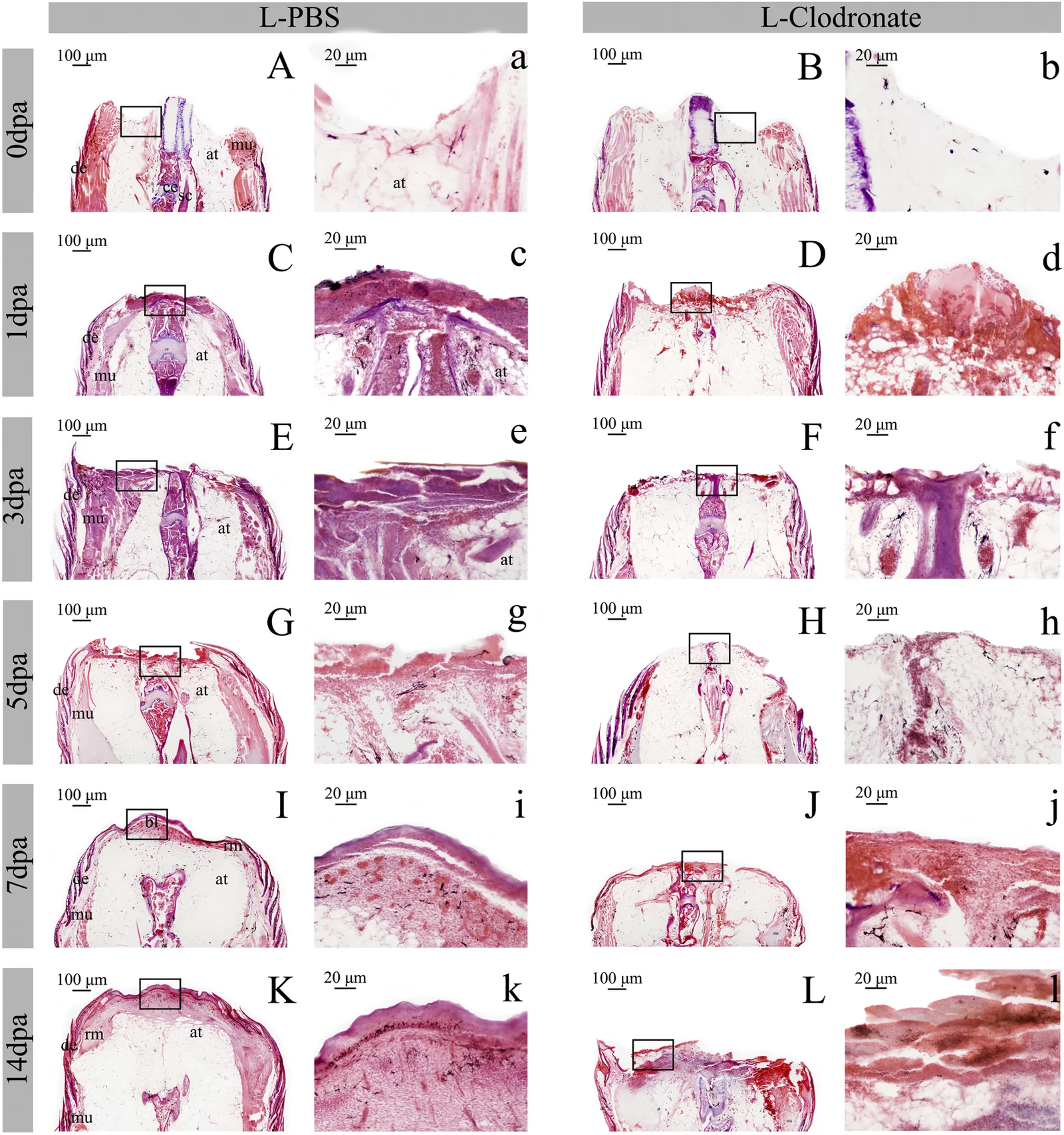
Tail regeneration morphology in *S. tsinlingensis* following L-Clodronate treatment. **(A–L)** Sagittal sections of regenerated tails. Scale bar, 100 μm. (a–l) High-magnification views of corresponding sections. Scale bar, 20 μm. Abbreviations: at, adipose tissue; bl, blastema; ce, central vertebra; de, dermis; mu, muscle; rm, regenerated muscle; sc, spinal cord; we, wound epithelium; cl, clot.

Histological analysis at 1 dpa showed spinal cord retraction and a blood clot covering the wound (Figures 2C,D). At 3 dpa, controls had reduced clot and an initiating wound epithelium (Figures 2E, e), whereas L-Clodronate animals retained a thicker clot (Figures 2F, f). At 5 dpa, the control epithelium thickened and sealed the wound; vertebral protrusion was resorbed (Figures 2G, g). In the depletion group, the epithelium remained thin and the wound stayed open (Figures 2H, h). By 7 dpa, controls formed a stratified wound epithelium, an apical epithelial cap (AEC) and a blastema (Figures 2I, i). The depletion group only achieved complete wound coverage by this stage, without AEC or blastema (Figures 2J, j). At 14 dpa, controls showed a thickened epithelium, a cartilaginous tube and substantial outgrowth (Figures 2K, k). The depletion group displayed disorganized epithelium, absence of AEC and stratification, and complete arrest of cartilage growth (Figures 2L, l).

### Phagocytic cell dynamics after depletion

**FIGURE 3:**
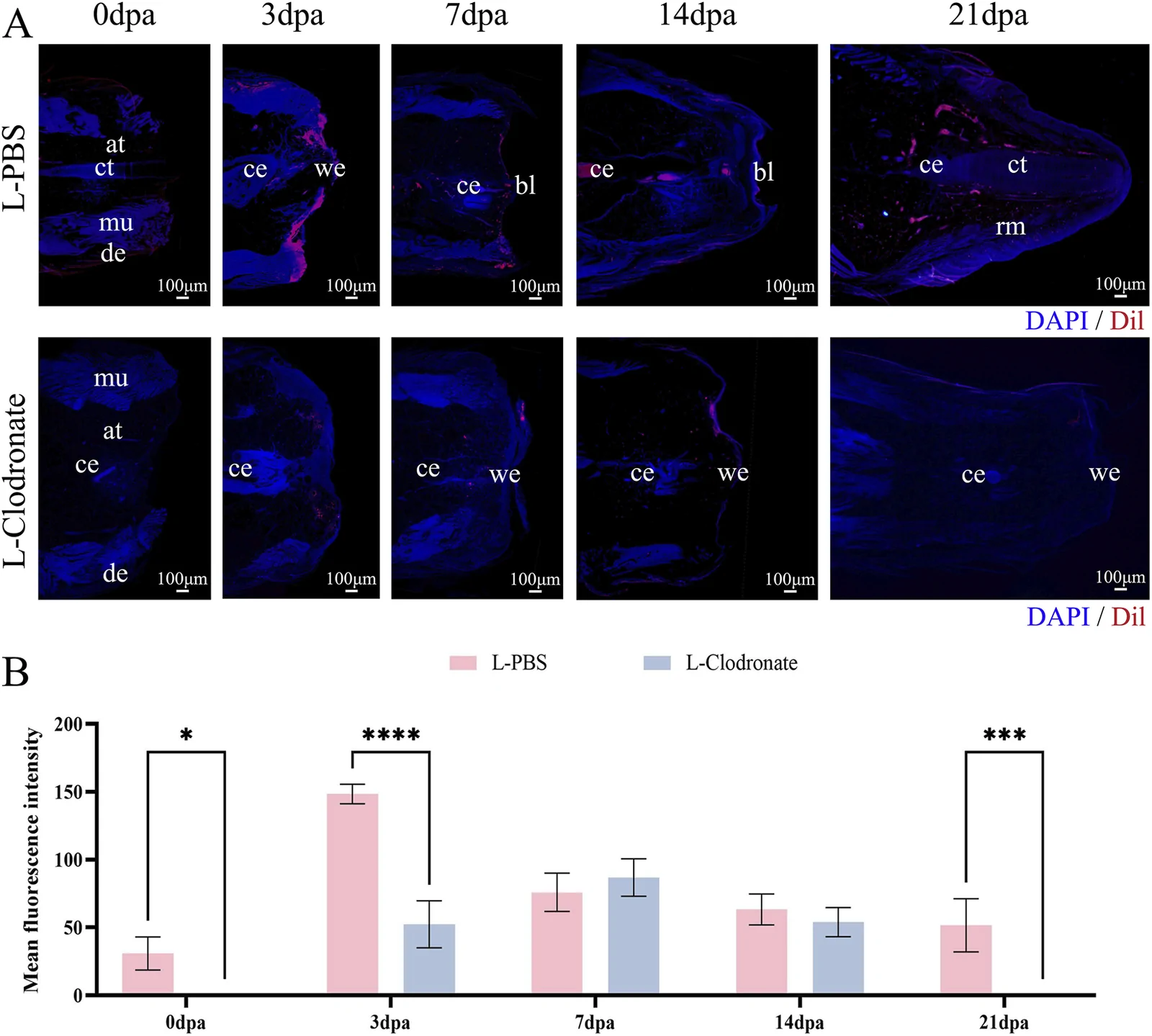
Dynamics of macrophage recruitment following tail amputation in *S. tsinlingensis*. **(A)** Histological analysis showing DiI-labeled macrophages (red) and DAPI-stained nuclei (blue) at indicated time points. Dashed line indicates the amputation plane. Scale bar, 100 μm. **(B)** Quantification of mean DiI fluorescence intensity (n = 3). *P < 0.05, P < 0.01, *P < 0.001, P < 0.0001.

DiI-labeled liposomes tracked macrophage recruitment. Control tails had few macrophages at 0 dpa. By 1 dpa, numerous macrophages accumulated subepidermally. At 3 dpa, controls showed strong macrophage labeling in the wound epithelium and spinal cord (mean fluorescence intensity 148.4 ± 7.1) compared to the depletion group (52.4 ± 17.4; P < 0.0001). Macrophage intensity in the depletion group at 3 dpa was reduced but not 0. At 7 dpa, control macrophage labeling had declined from its 3 dpa peak; the depletion group showed partial recovery (86.8 ± 13.8), which was similar to control levels at 7 dpa. In the L-Clodronate group, DiI + macrophages were detected only in adipose tissue and residual clot at 1-3 dpa, with a peak at 7 dpa (86.8 ± 13.8) that matched control levels at the same time point (Figures 3A,B). Mean DiI intensity in controls peaked at 3 dpa (148.4 ± 7.1) and gradually declined to 51.6 ± 19.6 by 21 dpa. In the depletion group, the peak occurred at 7 dpa (86.8 ± 13.8). At 14 dpa and 21 dpa, no statistically significant differences in macrophage intensity were observed between the two groups.

### CTSK^+^ osteoclasts mediate distal vertebral stump turnover

**FIGURE 4:**
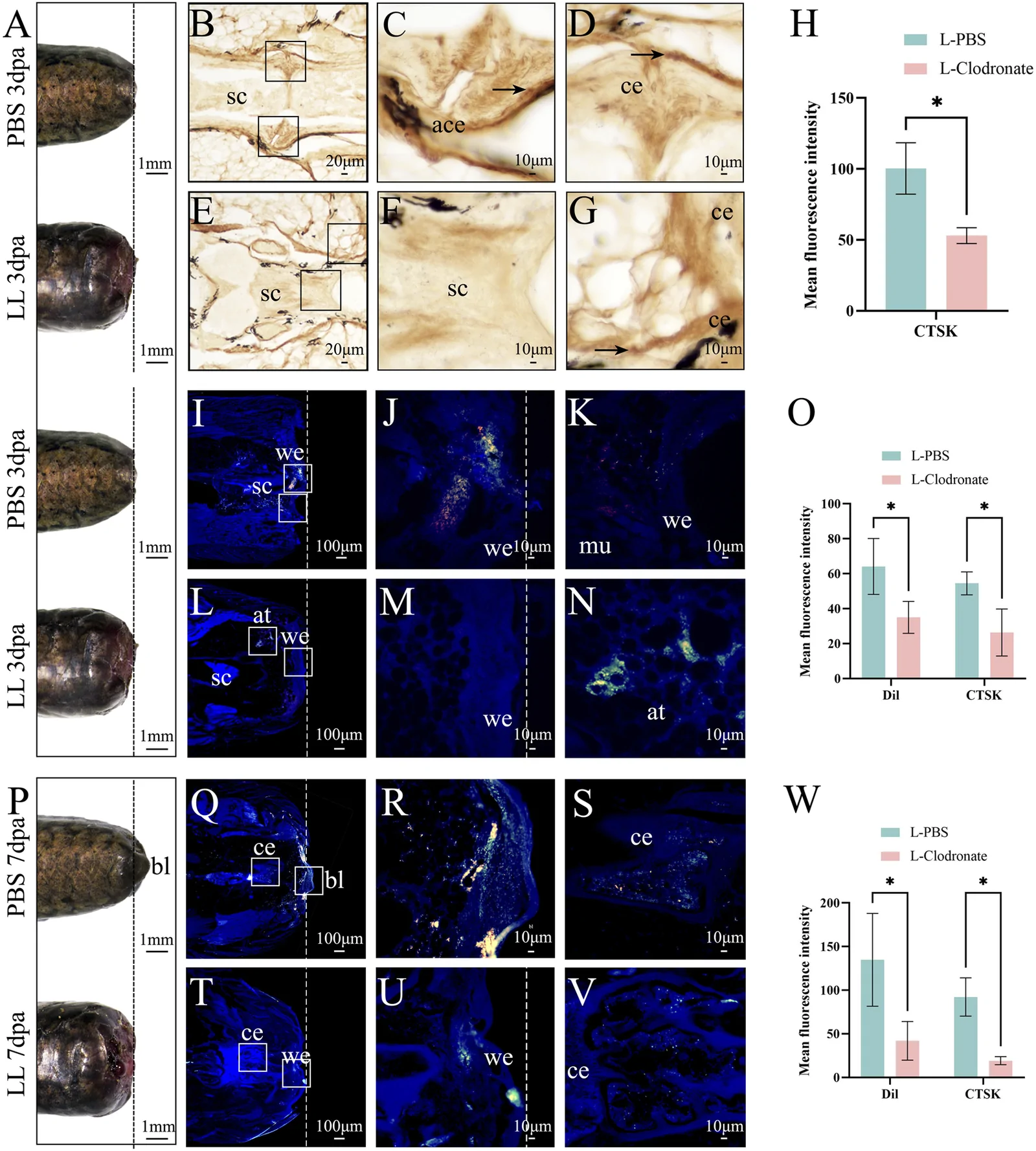
Clodronate-mediated depletion of CTSK^+^ phagocytic cells inhibits vertebral remodeling in *S. tsinlingensis* tail stumps. Morphological and immunohistochemical analysis at 3 dpa **(A–O)** and 7 dpa **(P–W)**. **(A,P)** Gross morphology of tails after L-PBS or L-Clodronate treatment. Scale bar, 1 mm. **(B,E,I,L,Q,T)** Sagittal sections stained for CTSK **(B,E)** or co-stained for CTSK (green) and DiI (red) **(I,L,Q,T)**. Scale bars: 20 μm **(B,E)**; 100 μm **(I,L,Q,T)**. **(C,D,F,G,J,K,M,N,R,S,U,V)** High-magnification views showing CTSK^+^ cell distribution in vertebral **(C,D)**, spinal cord **(F)**, epithelial **(G,J,K,M,R,U)**, adipose **(N)**, and blastemal **(S)** regions. Scale bar, 10 μm. **(H,O,W)** Quantification of CTSK^+^ cell numbers **(H)** and mean fluorescence intensity of CTSK/DiI **(O,W)**. Data are mean ± SEM; n = 3; *P < 0.05. Abbreviations: sc, spinal cord; we, wound epithelium; ce, central vertebra; ace, ablated central vertebra; at, adipose tissue; mu, muscle; bl, blastema.

Despite similar external morphology at 3 dpa (Figure 4A), internal ablation of the distal vertebral stump occurred only in controls, mediated by CTSK^+^ cells (Figure 4B). Controls had abundant CTSK^+^ cells (100.3 ± 18.1) associated with vertebral ablation (Figures 4C,D). The clodronate group had significantly fewer CTSK^+^ cells (53 ± 5.6; P < 0.05), which localized to the epithelium but not the spinal cord (Figures 4E–G,H). At 3 dpa, L-DiI/L-PBS controls showed DiI^+^/CTSK^+^ co-localization in the wound epithelium (Figures 4J,K); this was absent in the L-Clodronate group except in proximal adipose tissue (Figures 4M,N). Fluorescence intensities (DiI: 35 ± 9.1; CTSK: 26.3 ± 13.5) were significantly lower than in controls (DiI: 64.1 ± 16; CTSK: 54.5 ± 6.6; P < 0.05) (Figure 4O). At 7 dpa, controls showed strong co-localization in the epithelium/AEC (Figure 4R) and spinal cord (Figure 4S). The depletion group had weak epithelial signal (Figure 4U) and sporadic spinal distribution (Figure 4V), with significantly lower intensities (DiI: 41.9 ± 22.2; CTSK: 19.1 ± 4.6) than controls (DiI: 134.7 ± 53.2; CTSK: 92.1 ± 21.9; P < 0.05) (Figure 4W). Thus L-Clodronate effectively depleted recruited CTSK^+^ macrophages.

### Transcriptomic analysis at the re-epithelialization stage

**FIGURE 5:**
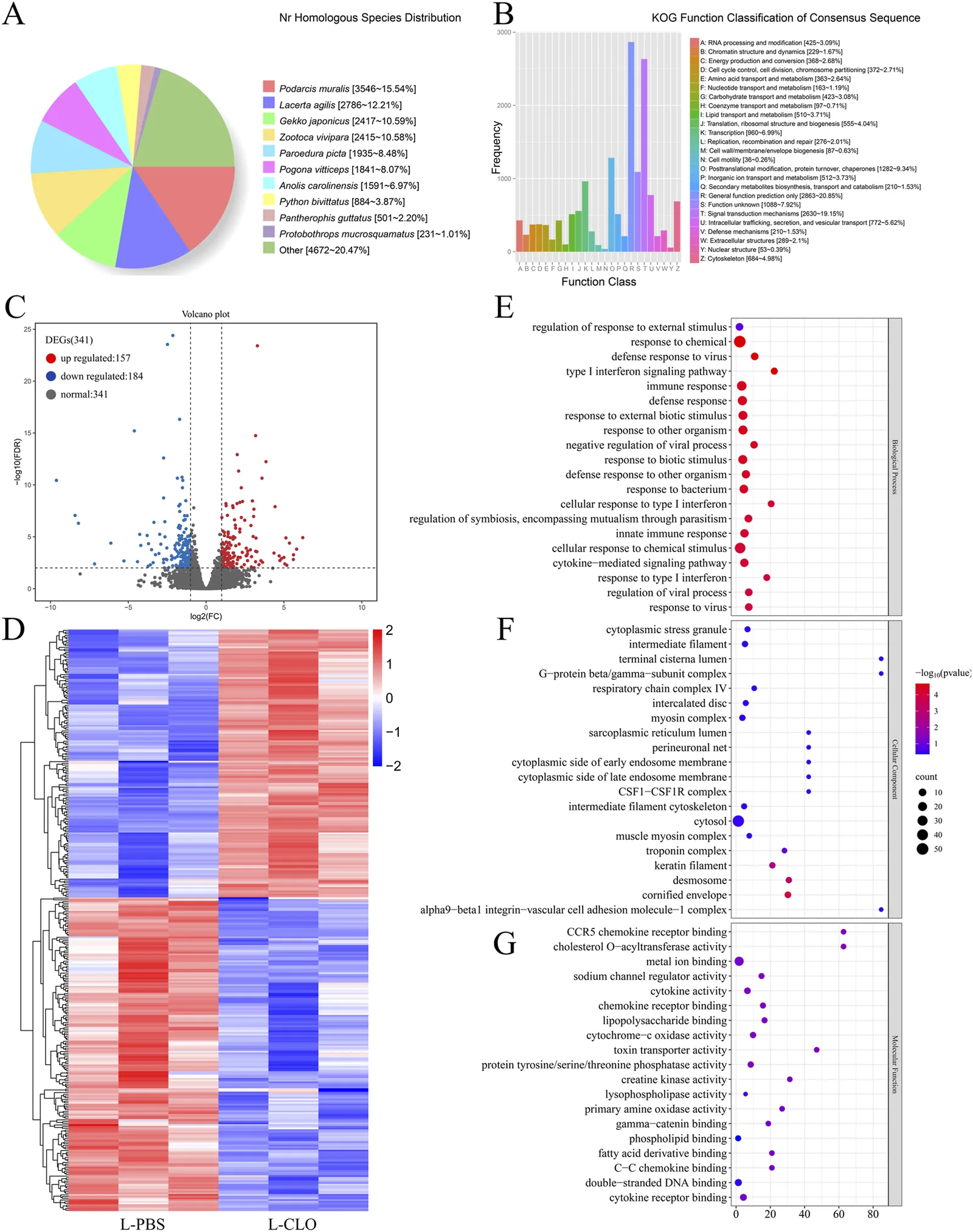
Transcriptome of *S. tsinlingensis* regenerating tail at 7 days post-PBS/CLO injection. **(A)** Species distribution of top BLASTx hits (Nr database). **(B)** KOG functional categories. **(C)** Volcano plot of DEGs (log_2_ FC vs. –log_10_FDR; red, upregulated; blue, downregulated). **(D)** Heatmap of DEG expression (PBS vs. CLO; red, high expression; blue, low expression). **(E–G)** GO enrichment (BP, CC, MF) shown as bubble plots (dot size, gene count; color, –log_10_P) with chord diagrams linking key GO terms to associated genes.

We compared regenerating tails at 7 dpa between L-PBS and L-Clodronate groups (n = 6 per group). Sequencing generated 36.12 Gb of clean data, with ≥5.81 Gb per sample and >98.09% of reads at Q30 (Supplementary Table S2). Assembly yielded 148,949 contigs (N50: 2,794 bp) and 90,943 unigenes (N50: 2,252 bp), of which 22,706 were >1 kb (Supplementary Table S3). A total of 24,823 were annotated in at least one database, including 22,819 in NR, 3,847 in COG, 19,448 in GO, 18,769 in KEGG, 13,733 in KOG, 16,828 in Pfam, 10,914 in Swissprot, 23,452 in TrEMBL and 20,631 in eggNOG (Supplementary Table S4). NR homology analysis showed highest matches to *P. muralis* (3,546 unigenes), then *Lacerta agilis* (2786), *G. japonicus* (2417), *Zootoca vivipara* (2415) and *Paroedura picta* (1935) (Figure 5A). KOG annotation placed 13,733 unigenes into 25 categories; the largest groups were general function (2863, 20.85%), signal transduction (2630, 19.15%) and post-translational modification (1,282, 9.34%) (Figure 5B).

**FIGURE 6:**
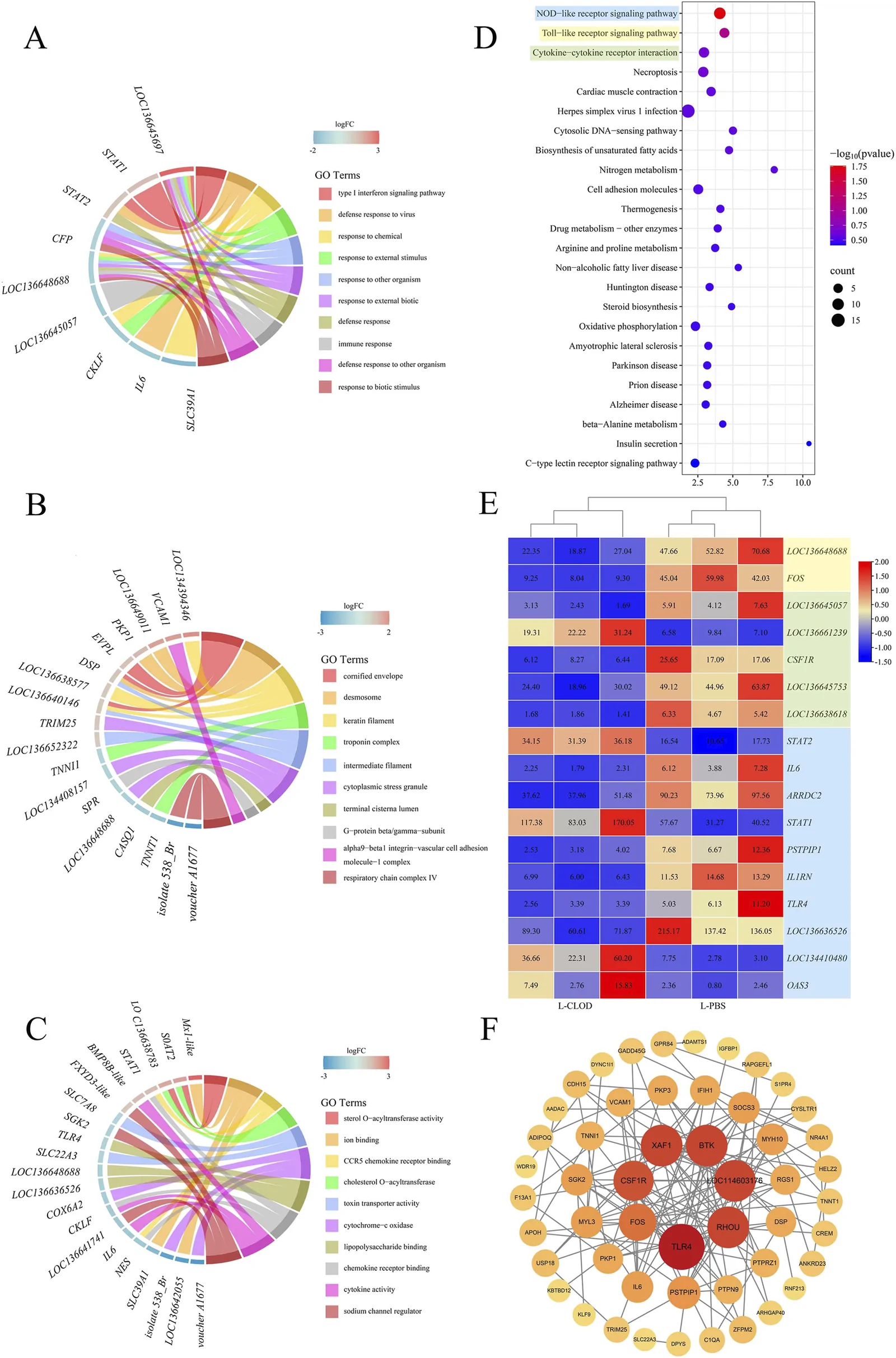
GO-based chord diagrams linking key terms to associated genes, KEGG enrichment, expression heatmap, and regulatory network in regenerating *S. tsinlingensis* tail at 7 dpa (L PBS vs. L Clodronate). **(A–C)** Chord diagrams for **(A)** Biological Process, **(B)** Cellular Component, and **(C)** Molecular Function, linking enriched GO terms to driving genes. **(D)** KEGG enrichment of 341 DEGs across 24 pathways, enriched in cytokine-cytokine receptor interaction, NOD-like receptor (NLR), and Toll-like receptor (TLR) signaling. **(E)** Heatmap of 17 DEGs overlapping among the three KEGG pathways in **(D)**. Right color blocks match pathway colors in **(D)** and indicate corresponding DEGs. **(F)** Multi-hub network after macrophage depletion. Core hubs (TLR4, CSF1R, BTK, XAF1, RHOU, FOS, IL6, PSTPIP1) form interconnected modules regulating immune/inflammatory (TLR/NF-κB, JAK-STAT), apoptosis (p53), and cytoskeletal remodeling (Rho GTPase, focal adhesion), coordinating inflammation, cell clearance, and tissue morphogenesis during tail regeneration.

We identified 341 differentially expressed genes (DEGs) between the L-PBS and L-Clodronate groups at 7 dpa (Figure 5C). The heatmap showed that more genes were downregulated than upregulated in the L-Clodronate group (Figure 5D). With the chord diagrams breaking each down (Figures 5A–C), GO enrichment analysis (Figures 5E–G; 6A–C; Supplementary Figure S1) showed that the downregulated DEGs fell into three functional categories. For biological process, they were enriched in type I interferon signaling (e.g., *STAT1*, *STAT2*), defense response to virus (including *STAT2* and *IL6*), and general immune response. For cellular component, the downregulated genes were associated with the cornified envelope (*EVPL*, *DSP*), desmosome (*PKP1*, *DSP*), keratin filament, and troponin complex (*TNNI1*). Regarding molecular function, they were enriched in chemokine receptor binding (*STAT1*, *CKLF*), cytokine activity (*IL6*, *CKLF*), sodium channel regulator activity, and CCR5 chemokine receptor binding (*STAT1*, *NES*).

KEGG analysis (Figure 6D) showed that the 341 DEGs were significantly enriched in cytokine-cytokine receptor interaction, NOD-like receptor (NLR) signaling and Toll-like receptor (TLR) signaling pathways. TLR4 emerged as a hub gene (Figure 6F). A heatmap of 17 overlapping DEGs among the three enriched KEGG pathways is shown in Figure 6E. A PPI network (50 nodes, 132 edges) revealed three functional modules: an immune-inflammatory module centered on TLR4 (including CSF1R, IL6, FOS, SOCS3, IFIH1); an apoptosis/cell-survival module with XAF1, TLR4, BTK, FOS; and a cytoskeletal remodeling/migration module centered on RHOU (with PSTPIP1, DSP, PKP1/3, MYL3, MYH10) (Figure 6F).

### qRT-PCR validation of key pathways

**FIGURE 7:**
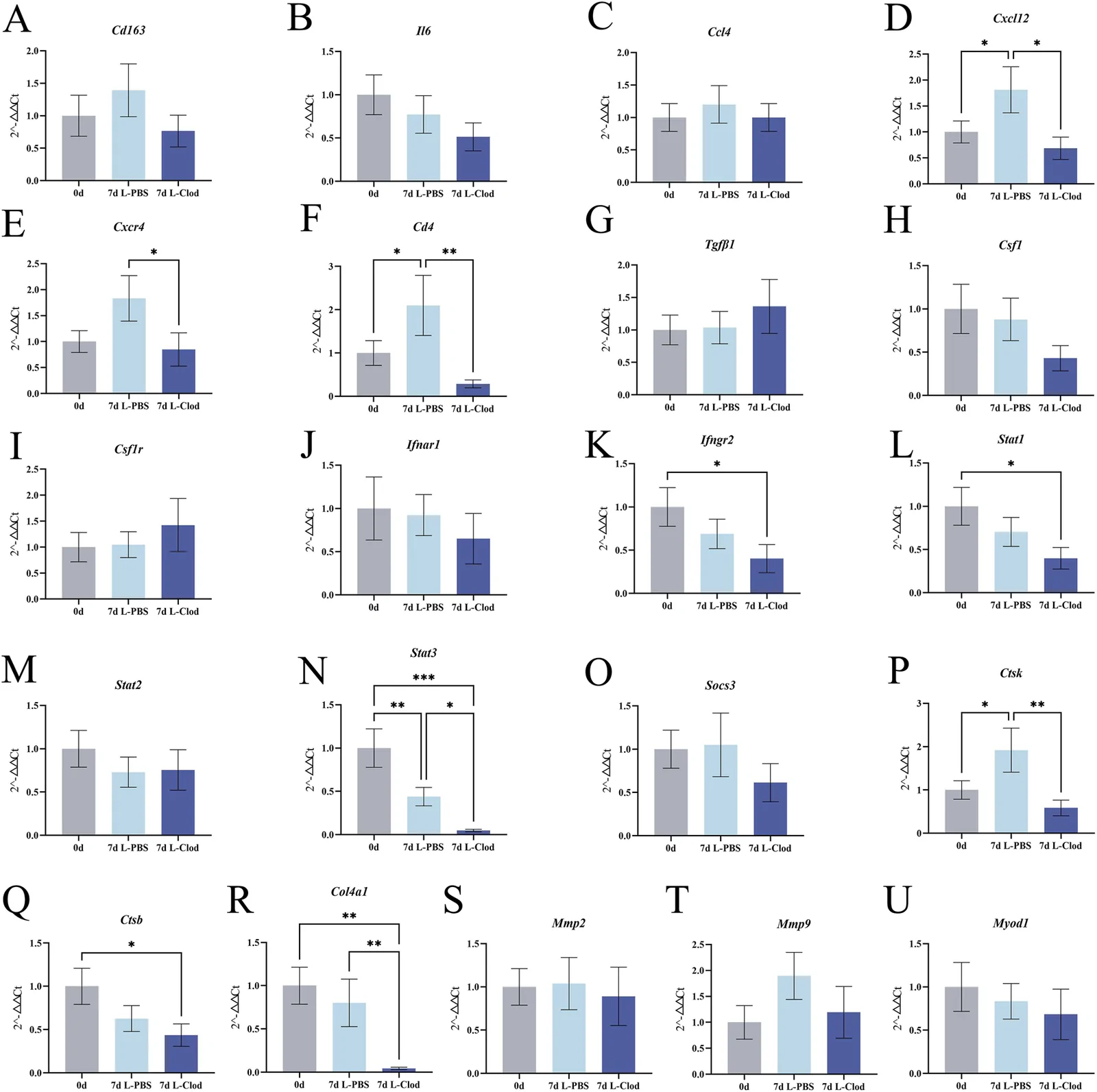
qRT-PCR validation of key pathways. **(A–G)** Immunomodulatory and inflammation-related molecules. **(H–O)** Macrophage homeostasis and signaling pathway. **(P–U)** Matrix remodeling-related molecules. The horizontal axis indicates days post-amputation under different treatments; the vertical axis (2^−ΔΔCt^) represents the relative expression ratio. n = 3. *P < 0.05, **P < 0.01, ***P < 0.001.

Quantitative PCR validated the transcriptomic changes for three categories of genes (Figure 7). Among immune-and inflammation-related genes, *Cd163* (an M2 macrophage marker) was downregulated in the depletion group, while *Il6* (pro-inflammatory and known to promote STAT3-mediated blastema formation) was also reduced. In contrast, *Tgfβ1* was upregulated in the depletion group, possibly acting as a compensatory fibrotic signal. The chemokine *Cxcl12* and its receptor *Cxcr4* were both lower at 7 dpa after macrophage ablation (P < 0.05), likely impairing stem cell homing. *Ccl4* showed a mild downregulation, and *Cd4*-elevated in controls at 7 dpa compared to 0 dpa (P < 0.05)-was suppressed in the L-Clodronate group. For macrophage homeostasis and signaling genes, *Csf1* was downregulated whereas *Csf1r* was upregulated at 7 dpa in the depletion group, suggesting possible compensatory expression. Interferon receptors *Ifnar1* (significantly) and *Ifngr2* (moderately) were downregulated. A discrepancy was noted for *Stat1/2*: qPCR indicated downregulation at 7 dpa but RNA-seq suggested upregulation, perhaps reflecting an early compensatory response that had not yet fully manifested at the mRNA level by that time. *Stat3* expression was significantly reduced, while *Il6* and *Socs3* showed non-significant decreases. Regarding ECM remodeling-associated genes, *Ctsk* was upregulated in controls at 7 dpa (versus 0 dpa) but markedly downregulated in the L-Clodronate group (P < 0.01); *Ctsb* followed a similar trend. *Col4a1*, a key basement membrane component, was strongly downregulated (P < 0.01), and both *Mmp2* and *Mmp9* were also reduced. Finally, *Myod1*, a driver of myogenic differentiation, decreased after macrophage depletion.

### Dysregulation of M1/M2 transition, cytokines, chemokines and oxidative stress after macrophage depletion

**FIGURE 8:**
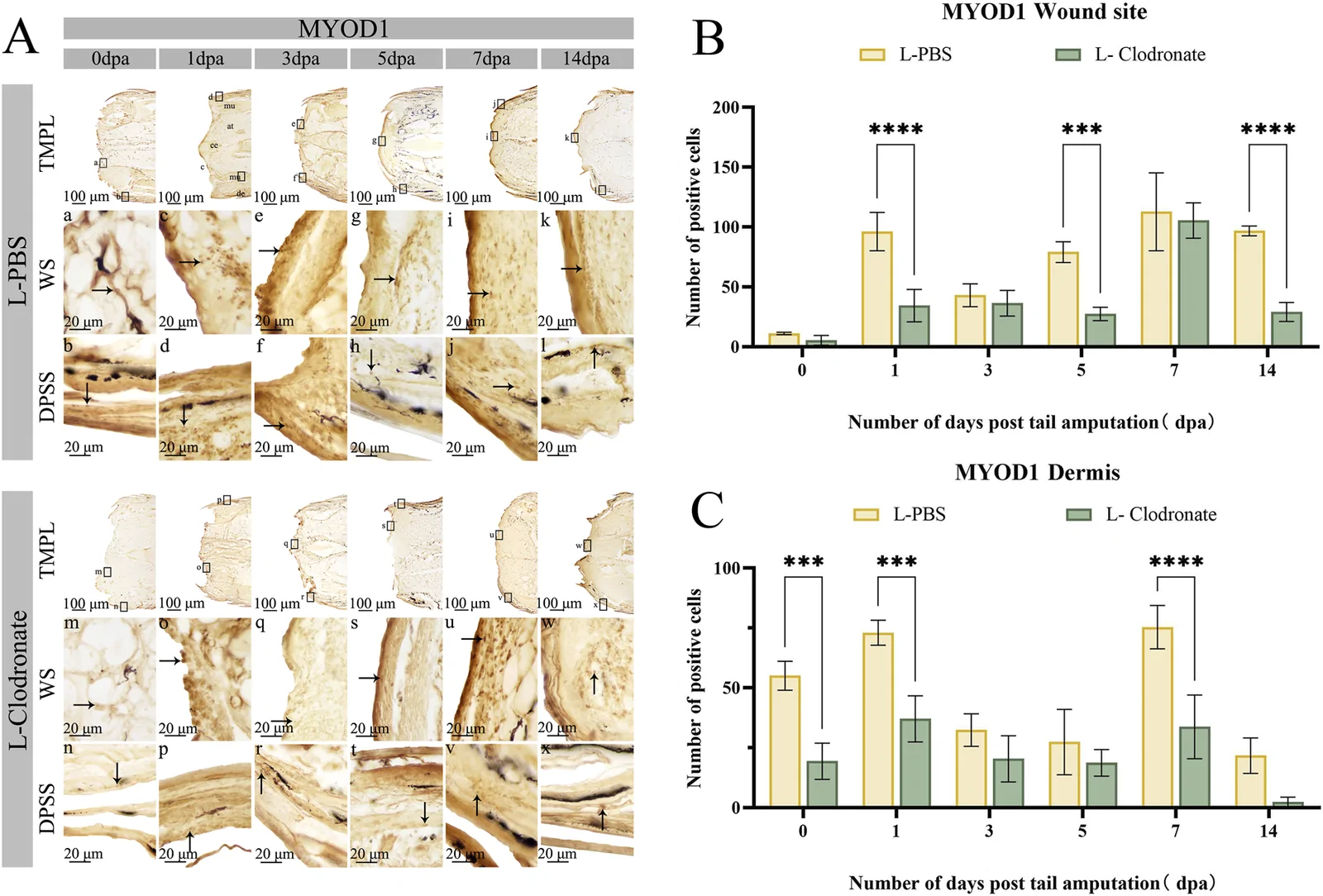
MYOD1 immunolocalization and quantification in *S. tsinlingensis*. **(A)** Immunolocalization of MYOD1 in L-PBS and L-Clodronate groups. **(B)** Quantification of MYOD1-positive cells at the wound site. **(C)** Quantification at the dermis site. Panels **,**(a, c, e, g, i, k) show the wound site; (b, d, f, h, j, l) show the dermal layer of scales (both 40×). Arrows indicate MYOD1-positive cells. Scale bar, 20 μm. Abbreviations: at, adipose tissue; de, dermis; mu, muscle; sc, spinal cord. n = 3. *P < 0.05, **P < 0.01, ***P < 0.001, ****P < 0.0001.

**FIGURE 9:**
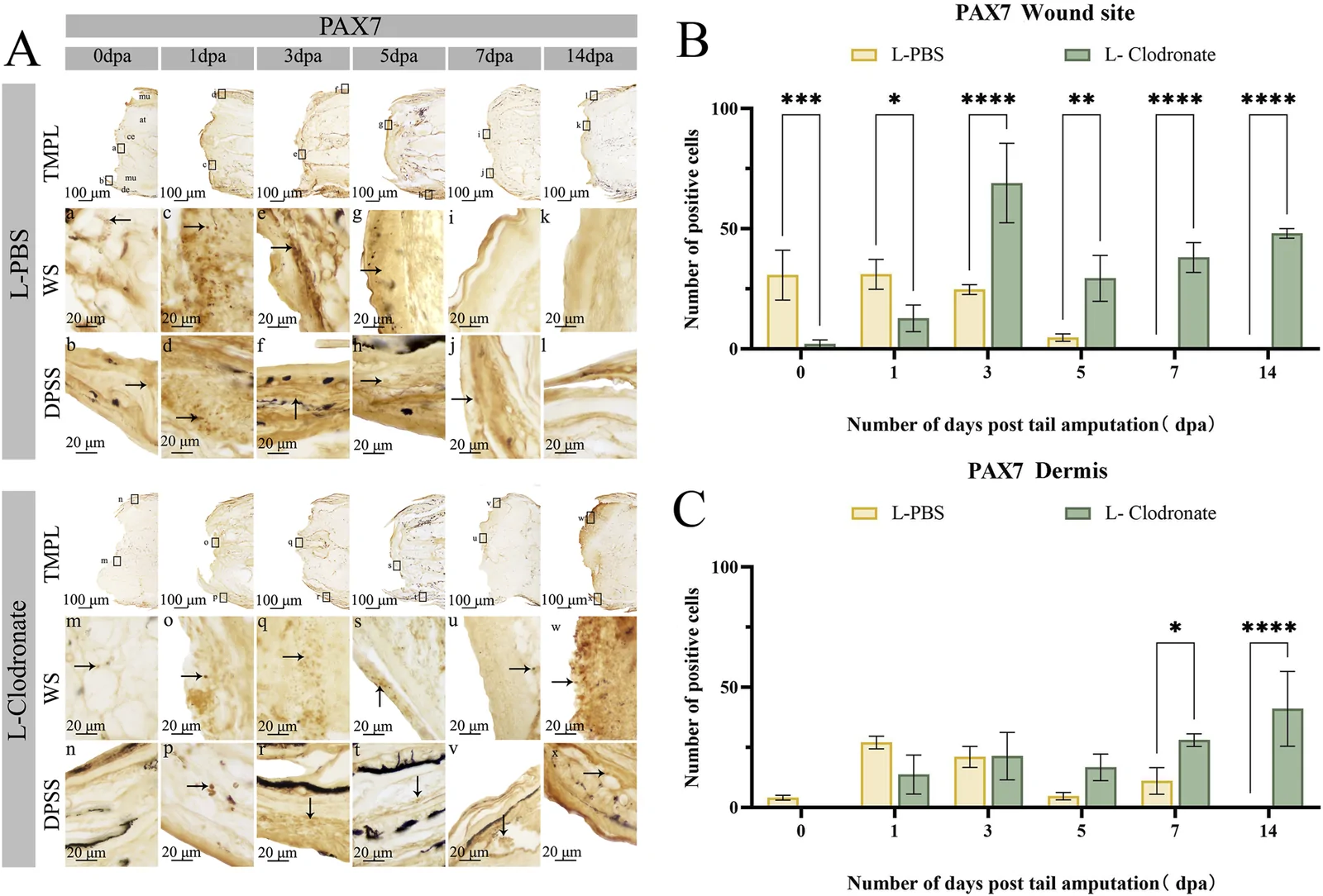
PAX7 immunolocalization and quantification in *S. tsinlingensis*. **(A)** Immunolocalization of PAX7 in L-PBS and L-Clodronate groups. **(B)** Quantification of PAX7-positive cells at the wound site. **(C)** Quantification at the dermis site. Panels (a, c, e, g, i, k) show the wound site; (b, d, f, h, j, l) show the dermal layer of scales (both 40×). Arrows indicate PAX7-positive cells. Scale bar, 20 μm. Abbreviations: at, adipose tissue; de, dermis; mu, muscle; sc, spinal cord. n = 3. *P < 0.05, **P < 0.01, ***P < 0.001, ****P < 0.0001.

We examined the spatiotemporal expression of key markers by immunohistochemistry at 0, 1, 3, 5, 7 and 14 dpa. Finally, myogenic markers showed profound disruption after macrophage depletion (Figures 8, 9). MYOD1 in controls exhibited two peaks: at 1 dpa (wound: 96.0 ± 16.0; dermis: 73.0 ± 5.2) and at 7 dpa (wound: 112.7 ± 32.6; dermis: 75.3 ± 9.1). Depletion significantly suppressed MYOD1 at both phases and failed to maintain expression by 14 dpa (wound: 29.0 ± 7.9 versus control 96.7 ± 4.0, P < 0.0001) (Figure 8). In contrast, PAX7 in controls peaked early (1 dpa: 31.0 ± 6.2) and then became undetectable, whereas L-Clodronate caused progressively increasing PAX7 levels that were significantly above controls from 3 dpa onward (3 dpa: 69.0 ± 16.5, P < 0.001; 14 dpa: 48.0 ± 2.0, P < 0.0001) (Figure 9).

**FIGURE 10:**
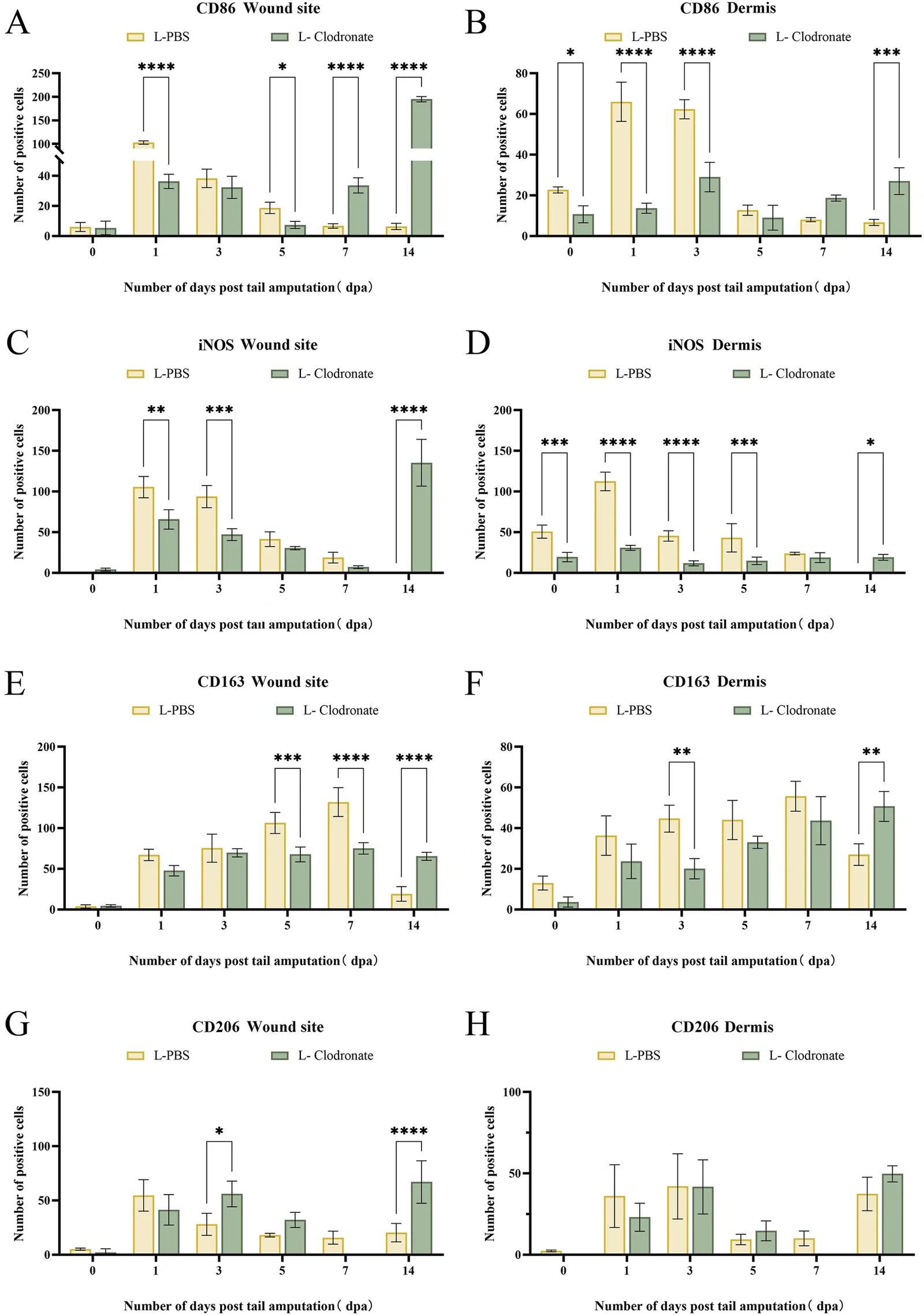
Expression of M1 and M2 macrophage markers in *S. tsinlingensis* tails after L-Clodronate vs. L-PBS treatment, quantified separately at wound and dermal sites. **(A,B)** CD86. **(C,D)** iNOS. **(E,F)** CD103. **(G,H)** CD206. Panels **(A,C,E,G)** show quantification at the wound site; panels **(B,D,F,H)** at the dermis site. n = 3. *P < 0.05, **P < 0.01, ***P < 0.001, ****P < 0.0001.

For M1 markers, control animals showed a peak of CD86^+^ cells at 1 dpa (wound: 102.7 ± 3.5; dermis: 66.0 ± 9.6) followed by a decline. L-Clodronate treatment strongly reduced early CD86 expression (1 dpa wound: 36.3 ± 4.7; P < 0.001) but caused a paradoxical rebound at 7–14 dpa, reaching 195.0 ± 5.6 at the wound site by 14 dpa, well above control levels (Figures 10A,B). iNOS followed a similar pattern: control peaked at 1 dpa (wound: 105.3 ± 13.1; dermis: 112.3 ± 11.4), depletion suppressed early iNOS but induced a rebound at 14 dpa (wound: 135.3 ± 28.7) (Figures 10C,D). Among M2 markers, CD163^+^ cells in controls peaked at 7 dpa (wound: 132.0 ± 17.7; dermis: 55.7 ± 7.4) then declined. Macrophage depletion suppressed CD163 at 7 dpa (wound: 75.0 ± 7.0, P < 0.0001) but caused sustained elevation at 14 dpa (wound: 65.3 ± 4.9, P < 0.01) (Figures 10E,F). CD206 peaked early in controls (1 dpa wound: 54.7 ± 14.5); depletion did not consistently suppress CD206 during inflammation but induced a late rebound at 14 dpa (wound: 67.0 ± 19.5 versus control 20.3 ± 8.5, P < 0.0001) (Figures 10G,H).

**FIGURE 11:**
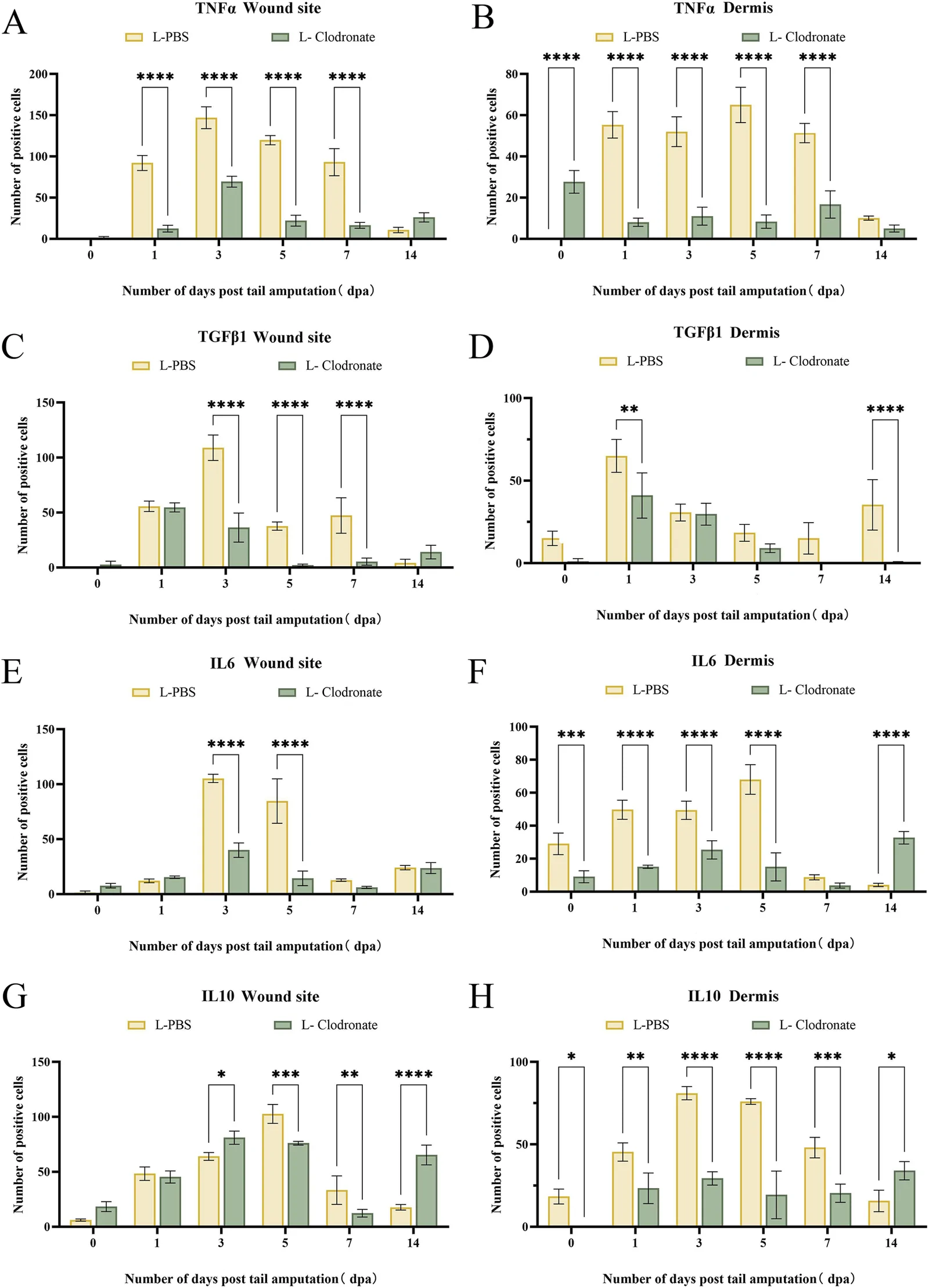
Expression of pro-inflammatory and anti-inflammatory factors in *S. tsinlingensis* tails after L-Clodronate vs. L-PBS treatment, quantified separately at wound and dermal sites. **(A,B)** TNFα. **(C,D)** TGFβ1. **(E,F)** IL6. **(G,H)** IL10. Panels **(A,C,E,G)** show quantification at the wound site; panels **(B,D,F,H)** at the dermis site. n = 3. *P < 0.05, **P < 0.01, ***P < 0.001, ****P < 0.0001.

Pro-and anti-inflammatory cytokines also showed distinct spatiotemporal changes (Figure 11). TNFα in controls peaked at 3 dpa in the wound (147.0 ± 13.2) and at 5 dpa in the dermis (65.0 ± 8.5); depletion suppressed TNFα throughout the inflammatory phase (3 dpa wound: 69.3 ± 6.7, P < 0.0001) (Figures 11A,B). TGF-β1 control levels peaked early (3 dpa wound: 109.0 ± 11.5) and were reduced by depletion (3 dpa wound: 36.3 ± 13.3, P < 0.001); by 14 dpa controls showed elevated dermal TGF-β1 while the depletion group remained low (Figures 11C,D). IL-6 in controls had a biphasic peak (3 dpa wound: 105.3 ± 3.8; 5 dpa dermis: 68.0 ± 9.0). Depletion suppressed early IL-6 but caused a rebound at 14 dpa (wound: 32.7 ± 3.8 versus control 4.0 ± 1.0, P < 0.0001) (Figures 11E,F). IL-10 control peaked in mid-inflammation (5 dpa wound: 102.7 ± 8.6; 3 dpa dermis: 81.0 ± 4.0); depletion suppressed early IL-10 but induced a rebound at 14 dpa (wound: 65.3 ± 9.0, P < 0.0001 versus control) (Figures 11G,H).

**FIGURE 12:**
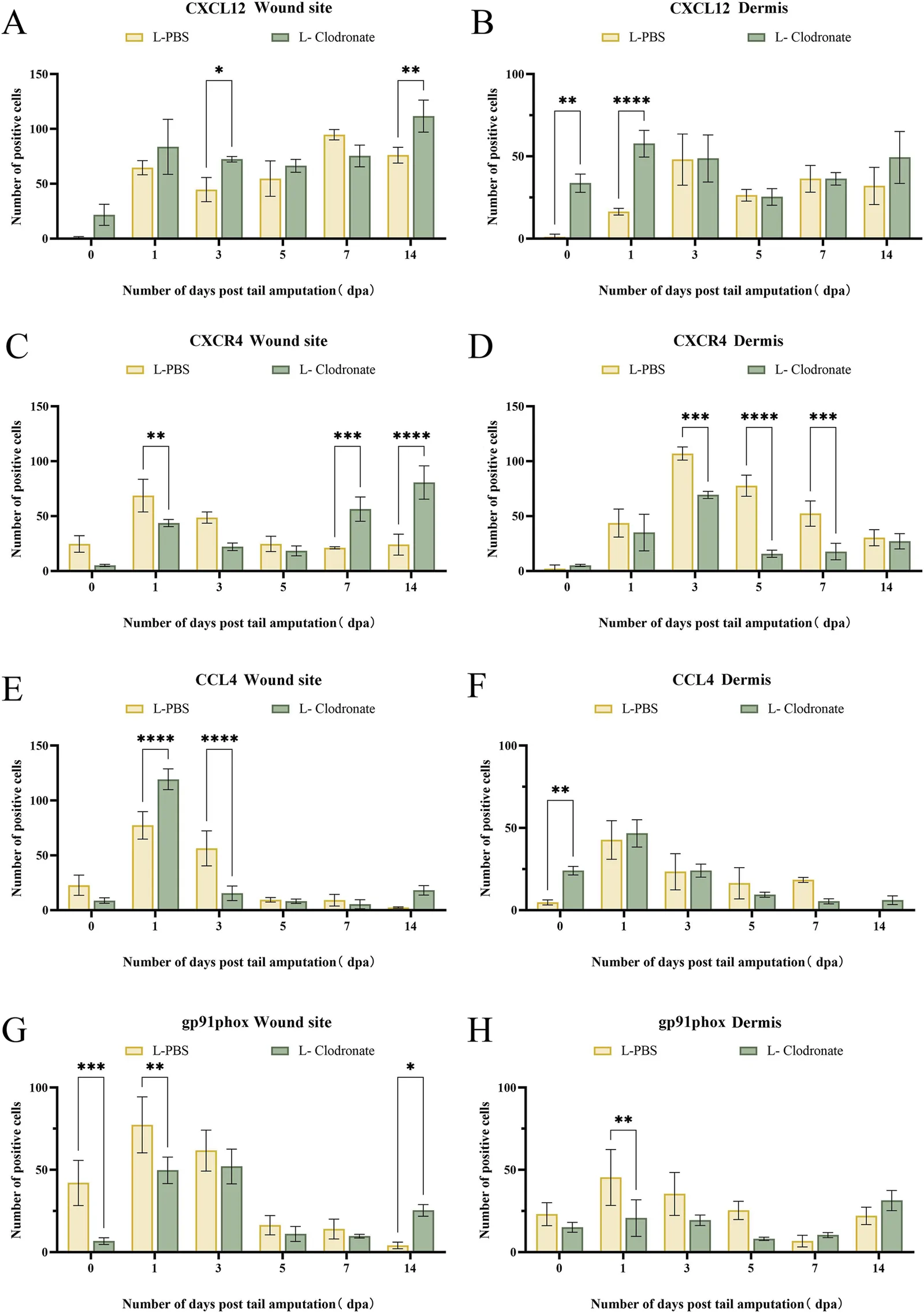
Expression of chemokines and oxidative stress factors in *S. tsinlingensis* tails after L-Clodronate vs. L-PBS treatment, quantified separately at wound and dermal sites. **(A,B)** CXCL12. **(C,D)** CXCR4. **(E,F)** CCL4. **(G,H)** gp91phox. Panels **(A,C,E,G)** show quantification at the wound site; panels **(B,D,F,H)** at the dermis site. n = 3. *P < 0.05, **P < 0.01, ***P < 0.001, ****P < 0.0001.

Chemokine expression was also dysregulated (Figures 12A–F). CXCL12 was elevated in the depletion group at several time points, including 3 dpa wound (72.3 ± 2.5 versus control 44.7 ± 11.0, P < 0.05) and 14 dpa wound (111.7 ± 14.6 versus control 76.0 ± 7.2, P < 0.0001) (Figures 12A,B). CXCR4 in controls peaked at 1 dpa in the wound (68.7 ± 14.8) and at 3 dpa in the dermis (107.0 ± 6.0); depletion reduced early CXCR4 but induced a rebound at 14 dpa (wound: 80.7 ± 15.2 versus control 21.0 ± 1.0, P < 0.001) (Figures 12C,D). CCL4 control peaked at 1 dpa (wound: 77.3 ± 12.5; dermis: 42.7 ± 11.7); depletion caused early enhancement (1 dpa wound: 119.3 ± 9.5, P < 0.01) followed by suppression and a late rebound (14 dpa wound: 18.0 ± 4.4) (Figures 12E,F). Oxidative stress, assessed by gp91phox, showed an early peak in controls (1 dpa wound: 77.3 ± 17.0; dermis: 45.3 ± 16.9) then declined; depletion suppressed early expression (1 dpa wound: 49.7 ± 8.1, P < 0.01) but caused a rebound at 14 dpa (wound: 25.3 ± 3.5 versus control 4.0 ± 2.0, P < 0.05) (Figures 12G,H).

### MMP inhibition delays but does not block tail regeneration

**FIGURE 13:**
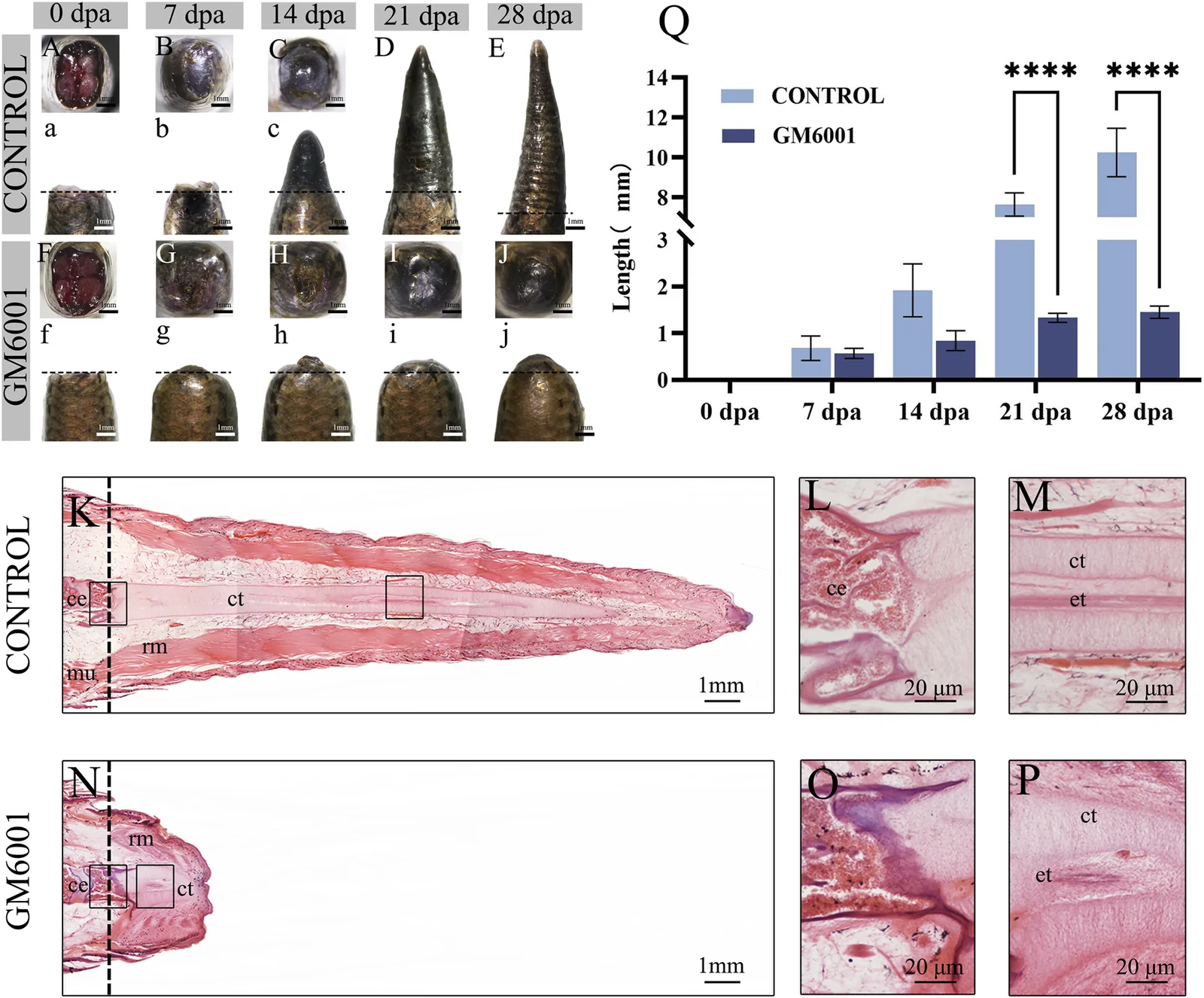
Inhibition of MMP activity by GM6001 disrupts tail regeneration in *S. tsinlingensis*. **(A–J)** Gross morphology after GM6001 or solvent injection. **(Q)** Quantification of regenerated tail length. **(K–P)** Histology (H&E) after 4 weeks. Sagittal sections of control **(K)** and GM6001-treated (N) tails. Scale bar, 1 mm. Boxed regions in controls **(L,M)** show organized muscle (rm) and ependymal tube (et), while GM6001 groups **(O,P)** display disorganized tissue and aberrant cartilage tube (ct). Scale bar, 20 μm. Abbreviations: ce, central vertebra; mu, muscle; sc, spinal cord.

GM6001 treatment severely slowed regenerative outgrowth over 4 weeks (Figure 13). At 7 dpa, controls showed wound epithelium thickening and small blastema (0.7 ± 0.3 mm), whereas GM6001 animals retained blood clots with delayed epithelialization. At 14 dpa, control blastemas reached 1.9 ± 0.6 mm; treated animals showed initial regeneration signs (0.8 ± 0.2 mm). At 21 dpa, controls measured 7.7 ± 0.6 mm vs. 1.3 ± 0.1 mm in the GM6001 group (P < 0.0001). At 28 dpa, the difference persisted (10.2 ± 1.2 mm vs. 1.5 ± 0.1 mm, P < 0.0001). Despite the delay, GM6001-treated tails eventually formed a cartilage tube, regenerated muscle and ependymal tube, indicating preserved regenerative capacity (Figures 13K–P).

**FIGURE 14:**
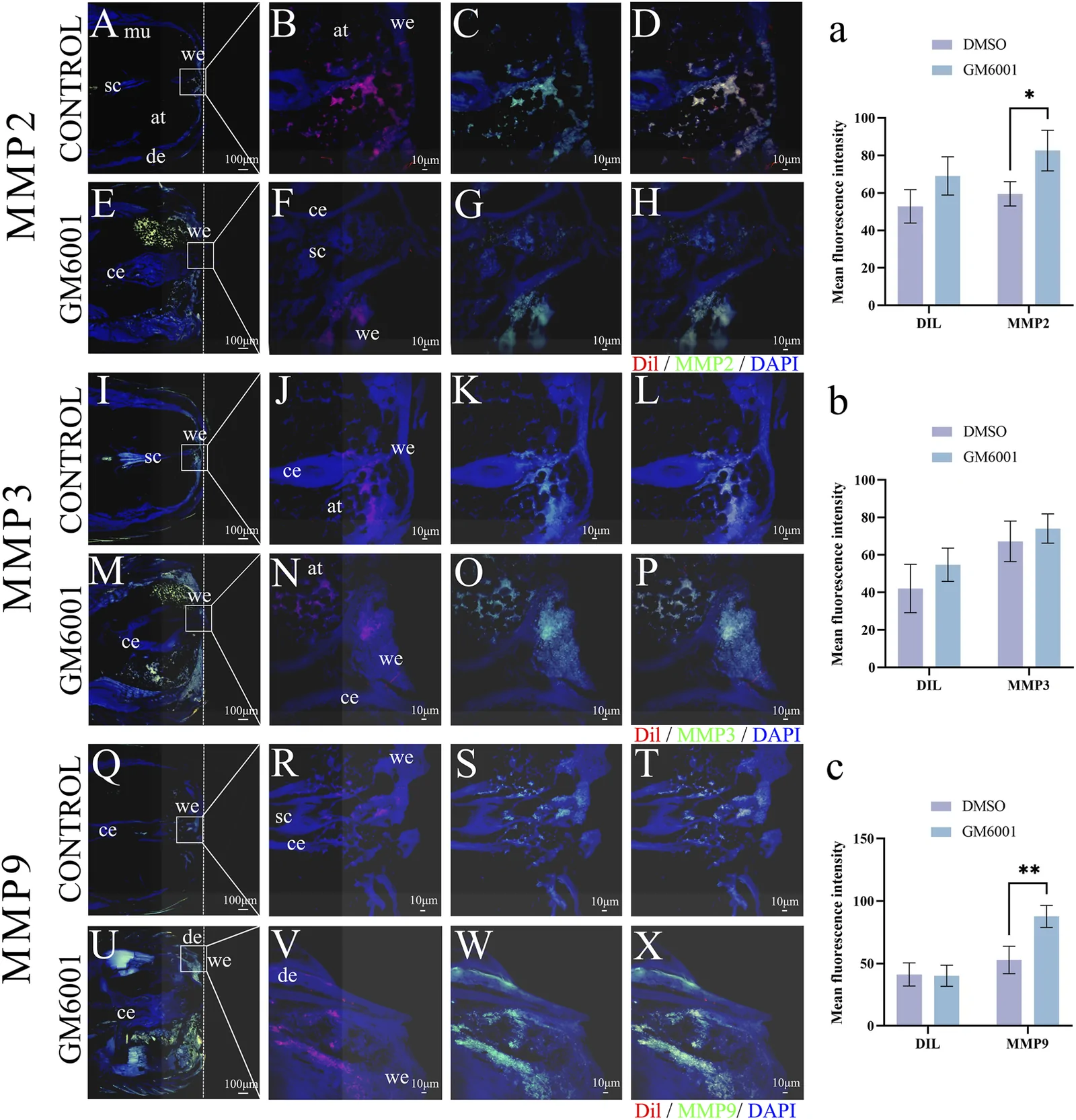
Distribution of matrix metalloproteinases in regenerating tails of *S. tsinlingensis* after 1 week of GM6001 injection. **(A–D)** Control group shows strong MMP2 expression (green) with extensive colocalization with DiI^+^ macrophages (red) in the wound epithelium. **(E–H)** GM6001 group exhibits disrupted MMP2 distribution and significantly reduced macrophage colocalization (n = 3, *P < 0.05). **(I–L)** Control group displays distinct MMP3 expression (green) colocalizing with macrophages (red). **(M–P)** GM6001 reduces MMP3 intensity without statistical significance. **(Q–T)** Control group demonstrates robust MMP9 expression (green) with macrophage (red) colocalization. **(U–X)** GM6001 substantially diminishes MMP9 expression and significantly impairs macrophage colocalization (P < 0.01). Scale bars: 100 μm (overview), 20 μm (magnified views). Abbreviations: we, wound epithelium; mu, muscle; sc, spinal cord; at, adipose tissue; de, dermis; ce, central vertebra.

Immunofluorescence at 7 dpa showed that GM6001 altered MMP distribution (Figure 14). Controls had MMP2 co-localization with macrophages in wound epithelium, spinal cord and adipose tissue (Figures 14A–D). GM6001 restricted co-localization to adipose tissue but significantly increased MMP2 fluorescence intensity (82.6 ± 10.8 vs. 59.5 ± 6.5, P < 0.05) (Figure 14A). MMP3 and macrophage co-localization in controls occurred in wound epithelium, adipose tissue and spinal cord (Figures 14I–L); inhibition eliminated spinal cord localization but maintained co-localization in adipose and epithelium (Figures 14M–P), with a non-significant increase in intensity (Figure 14B). The most striking effect was on MMP9: controls showed limited co-localization confined to the spinal cord (Figures 14Q–T), whereas GM6001 induced widespread MMP9 expression in muscle, adipose, wound epithelium and sub-scale dermis (Figures 14U–X), with significantly increased intensity (87.8 ± 8.8 vs. 52.9 ± 10.9, P < 0.05) (Figure 14C). Thus MMP inhibition prolongs MMP expression windows, likely as a compensatory response, and sustains macrophage presence, contributing to delayed but not absent regeneration.

## Discussion

Macrophages are required for tail regeneration in *S. tsinlingensis*. Systemic depletion with L-Clodronate caused complete failure of tail outgrowth within 21 dpa, with persistent hemorrhage, delayed re-epithelialization, absence of AEC and blastema, and a shift toward fibrotic repair. So macrophages are required not only in amphibians (Godwin et al., 2013) but also in a reptile, reinforcing the value of lizard tail regeneration as an instructive model for enhanced healing in adult amniotes (Lozito and Tuan, 2017).

Disrupted M1-to-M2 transition. In normal regeneration, M1 markers (CD86, iNOS) peaked during early inflammation (1–3 dpa), followed by M2 markers (CD163, CD206) at 7 dpa. This matches the classic M1→M2 switch that resolves inflammation and starts repair (Wynn and Vannella, 2016). Macrophage polarization into distinct M1/M2 phenotypes is a central determinant of repair versus fibrosis (Chen et al., 2019; Murray, 2017), and type 2 immunity, including M2 macrophages, plays key roles in tissue repair and fibrosis (Gieseck et al., 2018). Macrophage ablation completely broke this rhythm: early M1 was suppressed, then a paradoxical rebound of M1 occurred at 7–14 dpa, together with a late M2 surge at 14 dpa. These delayed and disorganized polarization events likely come from compensatory activation of tissue-resident or progenitor cells (Ginhoux and Guilliams, 2016), phenotypic reversal under chronic inflammation (Chen et al., 2023), or TLR/NF-κB signaling triggered by damage-associated molecules such as HMGB1 and ATP (Novak and Koh, 2013). In zebrafish, peripheral macrophages fine-tune inflammatory responses to promote regeneration (Morales and Allende, 2019). Yet the late M2 surge did not rescue blastema formation, so the timing of M2 signals—not just their presence—matters.

Cytokine dysregulation and failed compensation. In controls, TNFα showed two peaks (wound at 3 dpa, dermis at 5 dpa), consistent with roles in both inflammation and tissue remodeling (Chen and Nuñez, 2010; Aurora et al., 2014). TGFβ appeared early in the dermis (1 dpa), suggesting a role in necrotic debris clearance (Lodyga and Hinz, 2020). Depletion strongly reduced TGFβ, identifying macrophages as its main source; the later rebound of TGFβ in the depletion group (14 dpa) may instead promote scarring via TGFβ/Smad signaling (Wynn and Ramalingam, 2012). Persistent activation of macrophages and myofibroblasts, together with mechanical signals, drives fibrotic outcomes (Pakshir and Hinz, 2018). In lizards, repeated amputation or cauterization increases immune cell infiltration in blastemas and leads to scar-like healing (Alibardi, 2018). IL-6 and IL-10 were also misregulated. In controls, IL-6 helps M2 polarization via STAT3 (Seifert et al., 2012); its re-emergence at 14 dpa in depleted animals may reflect NF-κB-mediated compensation by blastema or AEC cells (Lv et al., 2017). The reduced expression of *Stat3* and *Socs3* in regenerating *S. tsinlingensis* tails likely resulted from *Il6* downregulation, which is known to suppress STAT3 activation and subsequently lower SOCS3 levels (Hoene et al., 2013). IL-10 normally resolves inflammation, but its sharp elevation at 14 dpa after depletion may reflect a delayed, maladaptive response that promotes fibrosis (Wynn and Ramalingam, 2012). Efficient wound repair requires coordinated actions of multiple cell types and signals (Eming et al., 2014).

Chemokine, redox and myogenic coordination. The CXCL12/CXCR4 axis was upregulated early (0–3 dpa) in controls, promoting stem cell homing and angiogenesis (Kucia et al., 2004; Ceradini et al., 2004), and again at 14 dpa, possibly maintaining blastema stemness via Wnt/β-catenin (Hu et al., 2013; Shan et al., 2015). Tissue-resident macrophages contribute to homeostasis and repair (Davies et al., 2013). Depletion caused early reduction of CXCL12/CXCR4 followed by a late rebound, but this rebound could not restore proper AEC formation, again showing that spatiotemporal precision matters more than absolute levels. CCL4 dynamics were also distorted, likely reflecting defective neutrophil recruitment (Kolaczkowska and Kubes, 2013; Shi and Pamer, 2011). Spatiotemporal analysis of gp91phox, a NADPH oxidase subunit, revealed disrupted redox dynamics upon macrophage depletion. Controls showed early gp91phox peaks at 1 dpa, where ROS from neutrophils and M1 macrophages cleared pathogens (Nathan and Cunningham-Bussel, 2013) and activated NF-κB/MAPK pathways to enhance inflammation (Mittal et al., 2014), while low-level ROS induced Nrf2-mediated antioxidant capacity (Tonelli et al., 2018). Levels subsequently dropped by 7-14 dpa, maintaining a pro-regenerative low-oxidative microenvironment (Love et al., 2013). Depletion suppressed early gp91phox due to lost M1 activity (Bedard and Krause, 2007), but induced late rebound at 14 dpa via NOX4 in blastema/fibroblasts (Hecker et al., 2009) or HMGB1-TLR signaling (Yang et al., 2020), potentially compensating via VEGF-induced angiogenesis (Ushio-Fukai and Nakamura, 2008) yet promoting fibrosis through TGFβ/Smad3 (Liu and Desai, 2015) and chronic inflammation via Treg suppression (Ohl and Tenbrock, 2018). Concurrently, myogenic markers revealed regeneration failure: control MYOD1 showed biphasic peaks (1 dpa and 7 dpa) for inflammation-initiated differentiation (Chargé and Rudnicki, 2004) and Wnt-mediated myoblast fusion (von Maltzahn et al., 2012), while depletion caused transient MYOD1 recovery followed by sharp decline, indicating impaired satellite cell activation (Serrano et al., 2008). PAX7 remained elevated in depleted groups, reflecting satellite cell “locking” in quiescence due to missing MYOD1-driven differentiation (von Maltzahn et al., 2012; Olguin and Olwin, 2004; Dumont et al., 2015). Macrophages thus coordinate regeneration through redox balance and myogenic regulationactivating MYOD1 via cytokines and balancing PAX7/MYOD1 through Notch-Wnt pathwayswhose ablation leads to compensatory but ineffective responses and regenerative failure.

Limitations and future directions. We used L-Dil and CTSK as broad markers for macrophages, without looking at finer subpopulations. Future single-cell RNA-seq should help characterize macrophage heterogeneity, including transitional M1/M2 states and tissue resident subsets, and how they contribute over time and space. Longer observation periods may also be needed to fully assess cartilage and nerve regeneration after macrophage depletion. It is still unclear whether the compensatory changes we saw, like the late M2 surge and upregulation of MMPs, could be used therapeutically to promote regeneration in non-regenerative settings. Our claim that TLR4 is a central regulator is based only on network connectivity; we would need functional validation to establish a causal role for TLR4 in tail regeneration. Morphological defects appeared well before 7 dpa, which makes it hard to tease apart the transcriptional effects of losing macrophages from those caused by impaired regeneration. Time-resolved or single-cell studies could help with that. Also, because we did RNA-seq on whole regenerates, the expression changes we saw could come from cell-autonomous regulation or from shifts in cell type composition. Single-cell transcriptomics would be needed to tell the difference.

## Conclusion

Macrophages are essential for tail regeneration in *S. tsinlingensis* by driving the M1-to-M2 phenotypic transition, coordinating cytokine and chemokine networks, maintaining redox balance, and enabling proper myogenic progression. Macrophage depletion disrupts these processes and triggers multiple compensatory responses, which nevertheless fail to restore regeneration within the 21-day observation period. In contrast, MMP inhibition delays but does not block regeneration, showing that macrophages act through a broader repertoire than ECM remodeling alone. Together, our histomorphological and transcriptomic data identify macrophages as spatiotemporal master regulators of lizard tail regeneration, and shed light on how regenerative programs evolve in vertebrates.

## Data Availability

The original contributions presented in the study are included in the article/Supplementary Material, further inquiries can be directed to the corresponding author.
