# Supplementary material for: Macrophages orchestrate tail regeneration in lizards Scincella tsinlingensis through spatiotemporal control of immunity, oxidative stress, and myogenesis
Source: Front Cell Dev Biol. 2026 Aug 6;14:1873713. doi: 10.3389/fcell.2026.1873713 (PMC13490066; doi:10.3389/fcell.2026.1873713)
Supplement: Supplementary file 1 [file DataSheet1.pdf]

Supplemental Information

Supplementary Table S1 Primer sequences used to amplify 22 genes.

Primer name	Forward sequence (5'-3')	Reverse sequence (5'-3')
Ifnar1	F: AGTGGCAGTGAGTCTTATG	R: ACATCTATTGGTGGTTCAGT
Ifngr2	F: GCCAAGAACAATCAGCAATG	R: CAATGTTGAGGGAGAATGATGA
Csf1	F: TCAACCTACTCCAGCCTATT	R: TGAAGCCTGAAGCCATTG
Csf1r	F: TCAACCTACTCCAGCCTATT	R: TGAAGCCTGAAGCCATTG
Cd163	F: CTCTGTCCGATTGCTGTTC	R: GTAGTGATGTGGTCTCTTGATG
Il6	F: ACTTCTCTGTTTCCTCTTCTCT	R: CCAATGCTGAGTTCTTCTTCT
Stat1	F: ACCACATCCCTTCCCATT	R: GCCAACTCAGCACATCAG
Stat2	F: CTTCCACCACCAGATGAG	R: TCCTCTTCAGTCACATTCAG
Stat3	F: CTGGCTGGACAACATCAT	R: ACCTTCTTTGCTGCTCTC
Socs3	F: GCAACACCAAACCTTTATCT	R: CAGCAGCATCAGTCTCTT
Tgfβ1	F: TCCTTGTTGCCTCACCTT	R: CTCCTCCCATCCATCTCTG
Cxcl12	F: ACTGGCAAGGGTTTCTCT	R: GGTCTTTCGGGTTCTCTTTC
Cxcr4	F: GGACCTGCGAATAAGTGG	R: GCTATGCTATGCTCAAGTGT
Ccl14	F: TGCCATCAGTTGTGTTTATCA	R: CTCTCCTCAGTTCCGAATCT
Myod1	F: GGGATTTCACTGTCGCTTA	R: AAGGATTCTGTTGGCTCAC
Ctsk	F: GCAGTCTTTCCAGTTCTACA	R: TAATCCAGTGCTTGCTTCC
Ctsb	F: TTCAAGACAGCAGAGGTG	R: AGCAACAAATGGGAGACA
Mmp2	F: CTTCCACCAACTTCCACTG	R: GACTGACTCGGCTTCTGA
Mmp9	F: TGGCTTCTCTGACTCTGA	R: GCATTGATGGGCATTACTG
Col4a1	F: GCAGAACAGGAAAGGCATT	R: GTAACCAGGCACAGTCAATC
Cd4	F: GGATAGGAGCAGAGATATGAC	R: CCGATTGAACCATAGGACTT
Gapdh	F: GCGTCTTCACCACCATAG	R: CTTGCCATTCCACCTGTT

Supplementary Table S2 Transcriptome sequencing statistics

Sample	Read Number	Base Number	GC Content	%≥Q30
d7-1	20,163,686	6,031,478,297	47.96%	98.40%
d7-2	19,423,971	5,807,853,398	48.25%	98.24%
d7-3	20,727,648	6,188,914,403	47.84%	98.19%
d7-CLO-1	20,486,272	6,121,185,680	47.54%	98.23%
d7-CLO-2	19,452,295	5,816,649,187	47.30%	98.09%
d7-CLO-3	20,609,282	6,151,275,264	47.58%	98.22%

Supplementary Table S3 Assembly statistics for *S. tsinlingenesis*

Length Range	Transcript	Unigene
200-300	33,933(22.78%)	29,305(32.22%)
300-500	28,085(18.86%)	21,161(23.27%)
500-1000	28,486(19.12%)	17,771(19.54%)
1000-2000	24,814(16.66%)	10,363(11.40%)
2000+	33,619(22.57%)	12,343(13.57%)
Total Number	148,949	90,943
Total Length	204,809,774	90,604,533
N50 Length	2,794	2,252
Mean Length	1375.03	996.28

Supplementary Table S4 Results of unigene annotation

Anno Database	Annotated Number	Length (bp) 300-1000	Length (bp) ≥1000
COG_Annotation	3,847	460	3,241
GO_Annotation	19,448	4,263	13,239
KEGG_Annotation	18,769	4,060	12,770
KOG_Annotation	13,733	2,392	10,211
Pfam_Annotation	16,828	3,179	12,466
Swissprot_Annotation	10,914	1,751	8,477
TrEMBL_Annotation	23,452	5,781	14,772
eggNOG_Annotation	20,631	4,899	13,446
nr_Annotation	22,819	5,468	14,650
All_Annotated	24,823	6,542	14,977

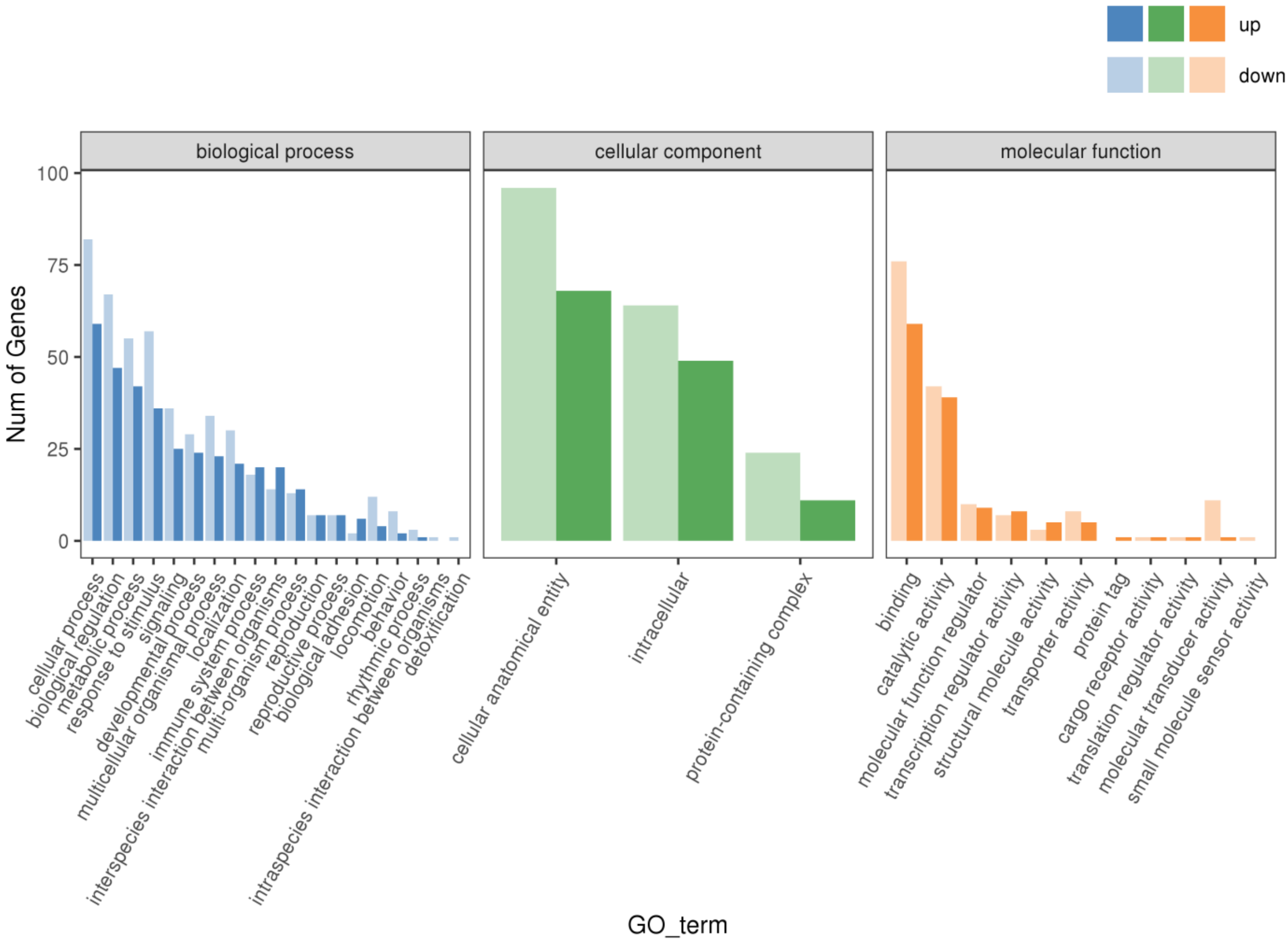

Supplementary Figure S1 GO enrichment map comparing L-PBS and L-Clodronate groups in regenerating *S. tsinlingensis* tails at 7 dpa.

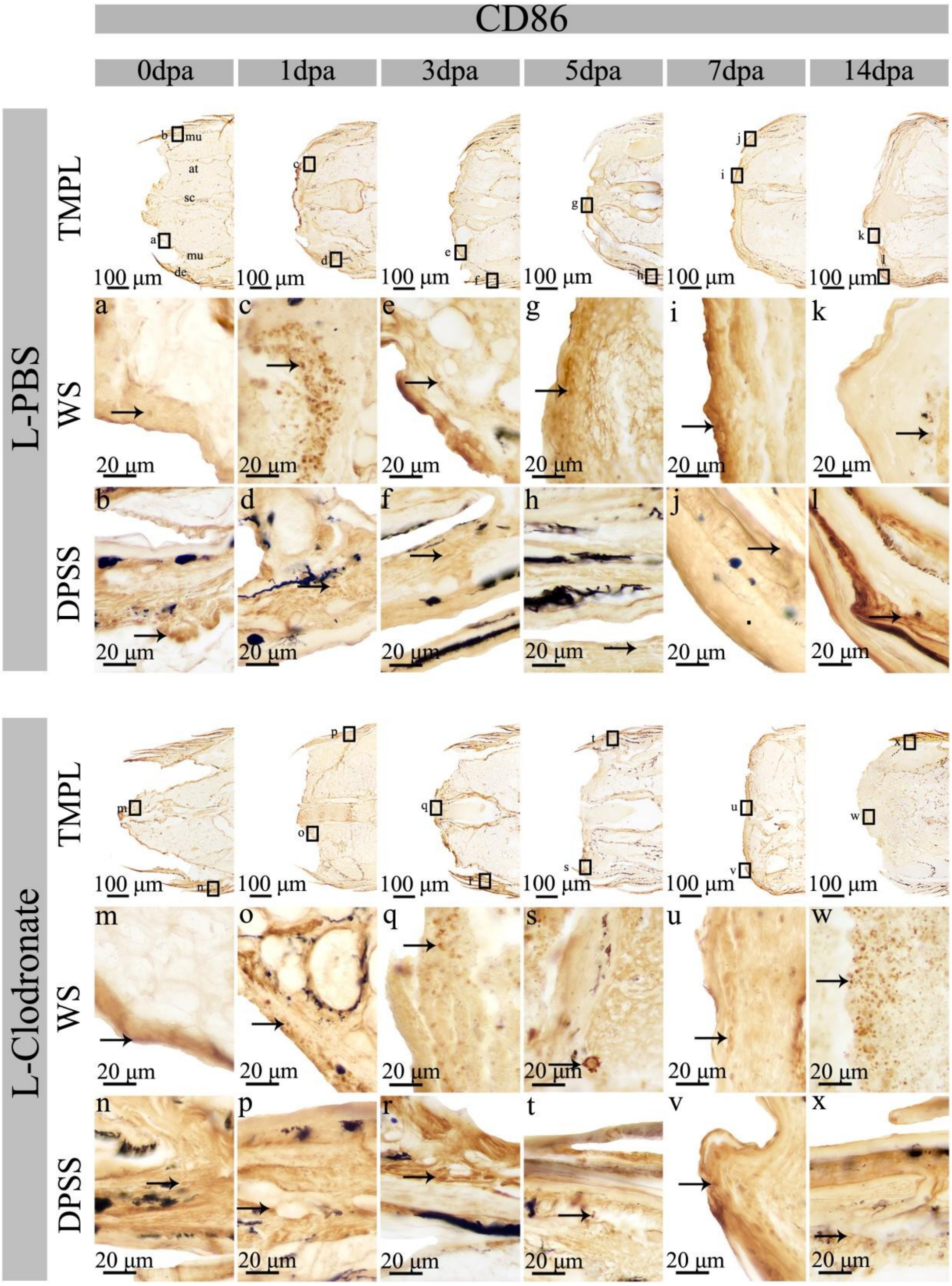

Supplementary Figure S2 Immunolocalization of CD86 in M1 macrophages of *S. tsinlingensis*: a comparative study of L-PBS and L-Clodronate treatment.

Note: Panels (a, c, e, g, i, and k) show the wound site under 40× magnification; Panels (b, d, f, h, j, and l) show the dermal layer of scales under 40× magnification. Arrows indicate CD86-positive cells. at. Adipose tissue; de. Dermis; mu. Muscle; sc. Spinal cord; TMPL:The tissue morphology and positive labeling; WS:Wound site; DPS: The dermis of proximal scale to the stump surface, bar = 20 µm.

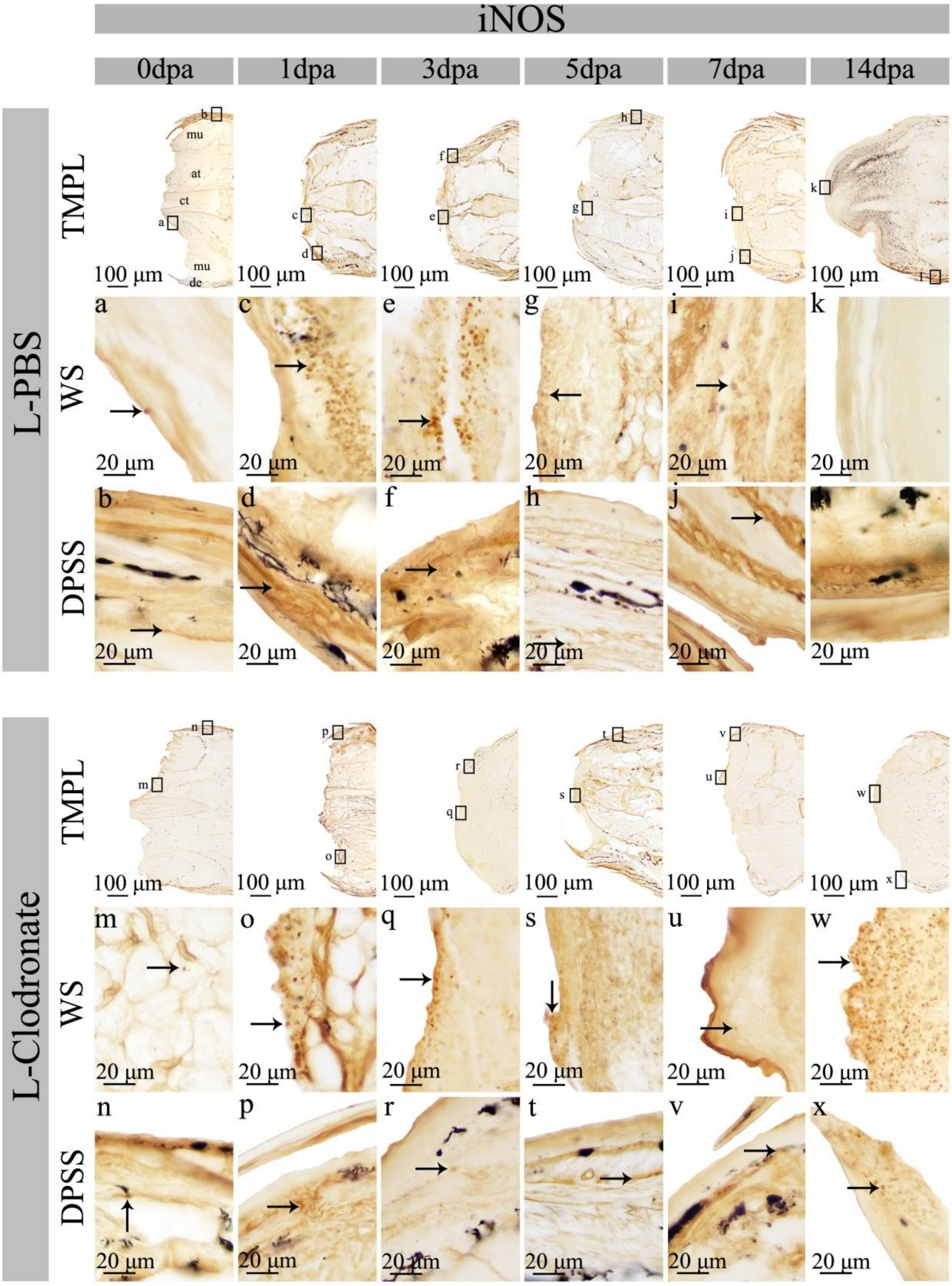

Supplementary Figure S3 Immunolocalization of iNOS in M1 macrophages of S. tsinlingensis: a comparative study of L-PBS and L-Clodronate treatment

Note: Panels (a, c, e, g, i, and k) show the wound site under 40× magnification; Panels (b, d, f, h, j, and l) show the dermal layer of scales under 40× magnification. Arrows indicate iNOS-positive cells. at. Adipose tissue; de. Dermis; mu. Muscle; sc. Spinal cord; TMPL: The tissue morphology and positive labeling; WS:Wound site; DPS: The dermis of proximal scale to the stump surface, bar = 20 μm.

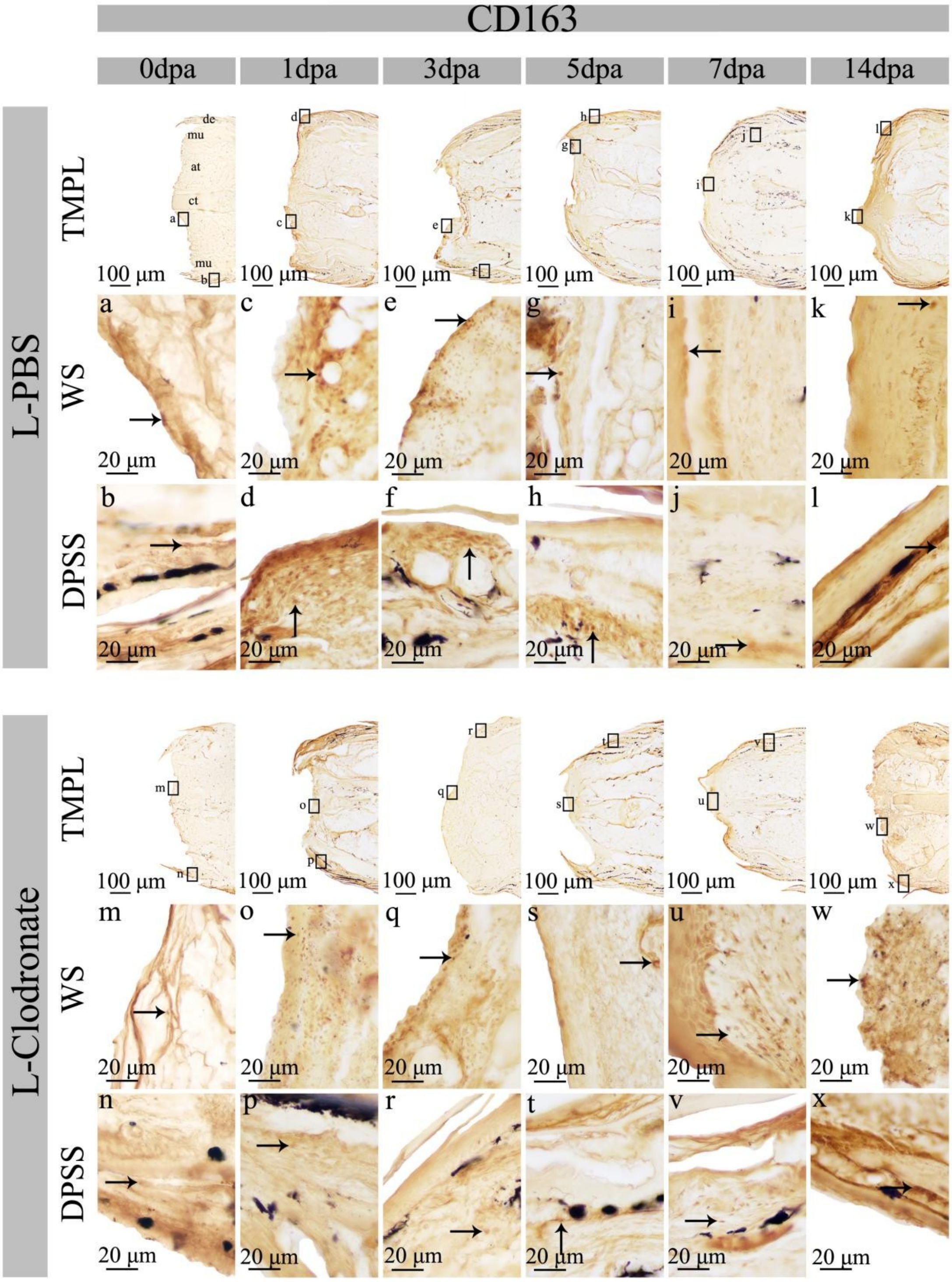

Supplementary Figure S4 Immunolocalization of CD163 in M2 macrophages of *S. tsinlingensis*: a comparative study of L-PBS and L-Clodronate treatment

Note: Panels (a, c, e, g, i, and k) show the wound site under 40× magnification; Panels (b, d, f, h, j, and l) show the dermal layer of scales under 40× magnification. Arrows indicate CD163-positive cells. at. Adipose tissue; de. Dermis; mu. Muscle; sc. Spinal cord; TMPL: The tissue morphology and positive labeling; WS:Wound site; DPS: The dermis of proximal scale to the stump surface, bar = 20 μm.

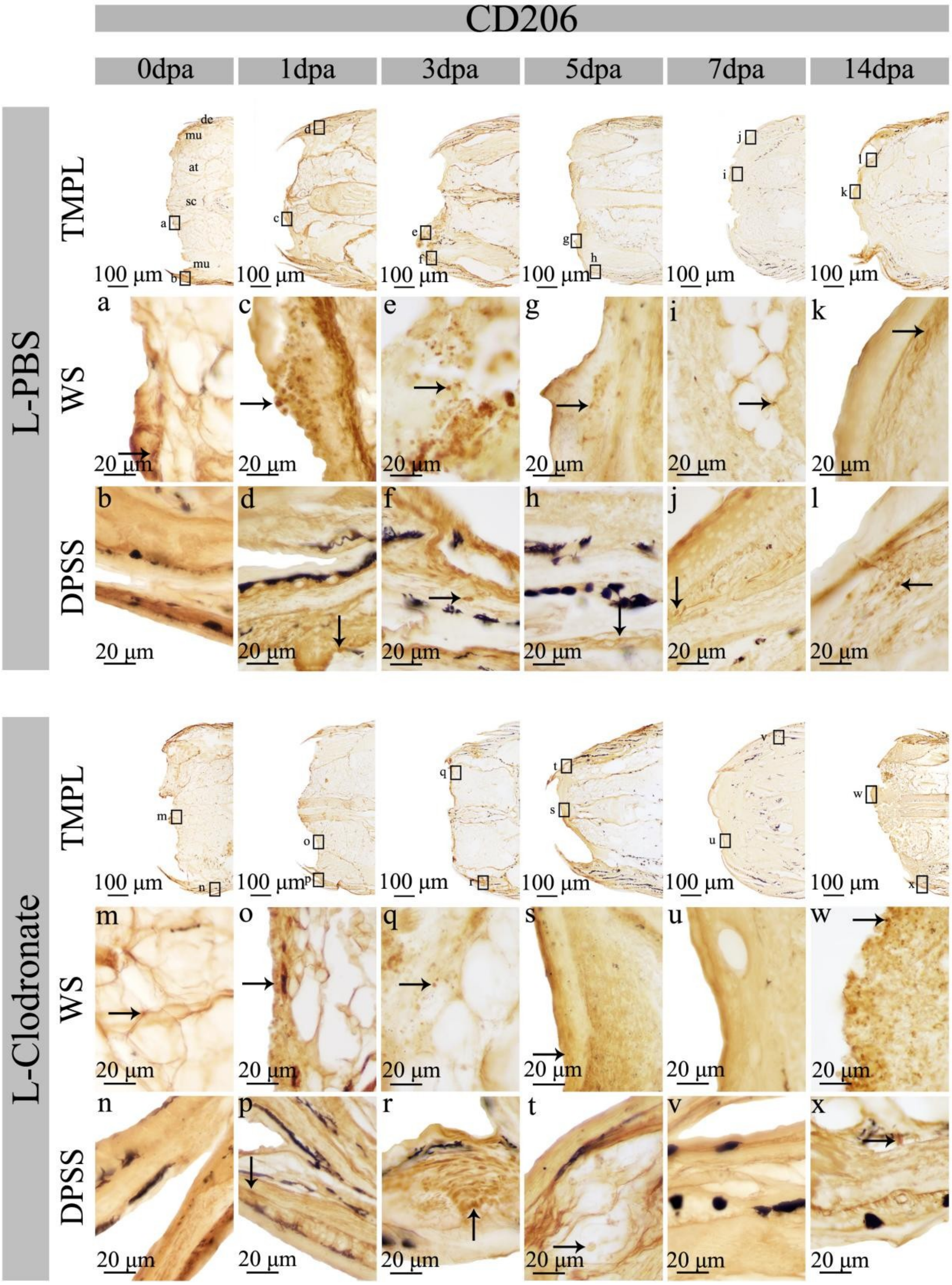

Supplementary Figure S5 Immunolocalization of CD206 in M2 macrophages of *S. tsinlingensis*: a comparative study of L-PBS and L-Clodronate treatment

Note: Panels (a, c, e, g, i, and k) show the wound site under 40× magnification; Panels (b, d, f, h, j, and l) show the dermal layer of scales under 40× magnification. Arrows indicate CD 206-positive cells. at. Adipose tissue; de. Dermis; mu. Muscle; sc. Spinal cord; TMPL: The tissue morphology and positive labeling; WS:Wound site; DPS: The dermis of proximal scale to the stump surface, bar = 20 μm.

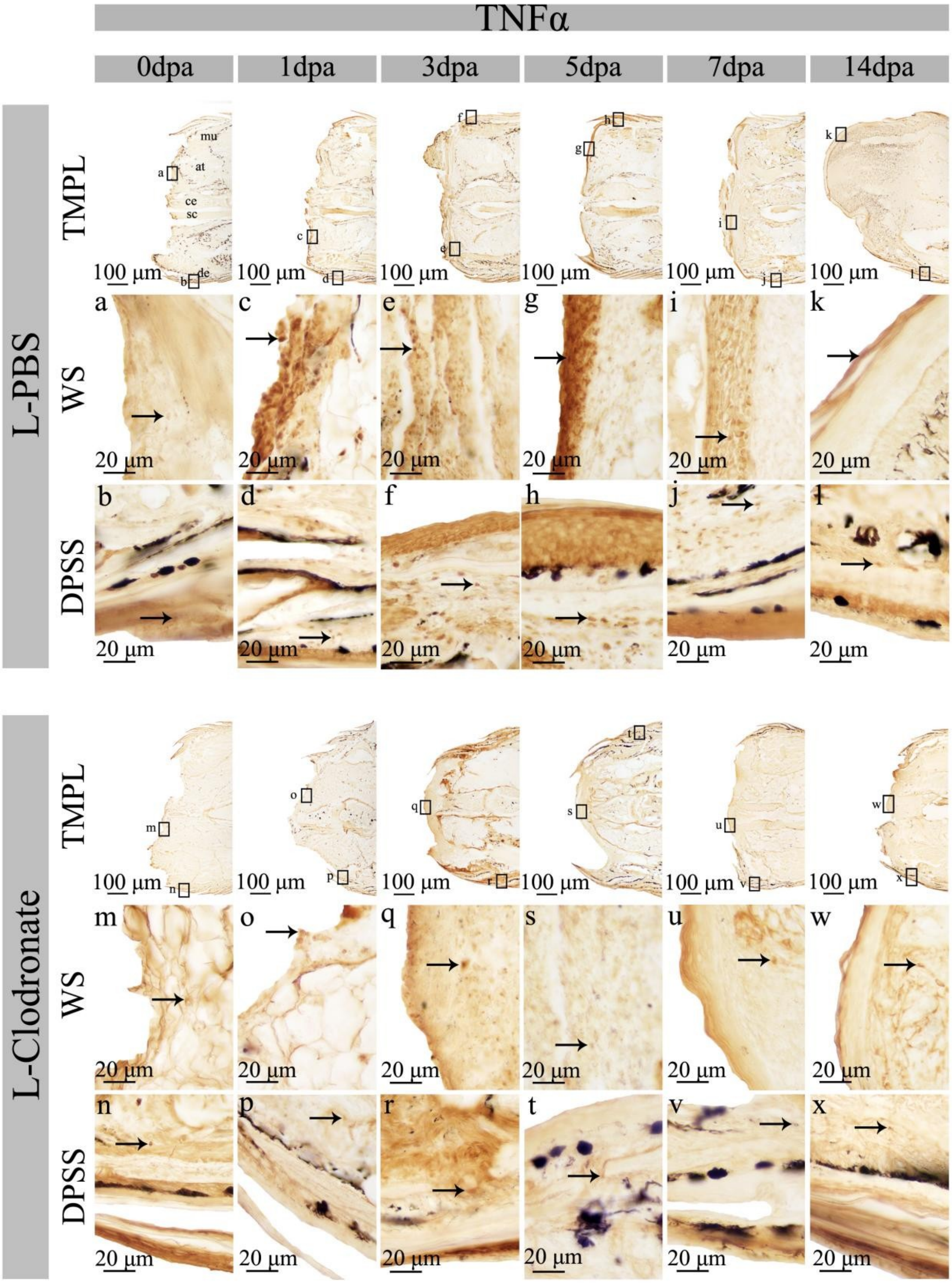

Supplementary Figure S6 Immunohistochemical localization of the pro-inflammatory factor TNFα in the L-PBS group and L-Clodronate group of *S. tsinlingensis*

Note: Panels (a, c, e, g, i, and k) show the wound site under 40× magnification; Panels (b, d, f, h, j, and l) show the dermal layer of scales under 40× magnification. Arrows indicate TNFα-positive cells. at. Adipose tissue; de. Dermis; mu. Muscle; sc. Spinal cord; TMPL: The tissue morphology and positive labeling; WS:Wound site; DPS: The dermis of proximal scale to the stump surface, bar = 20 μm.

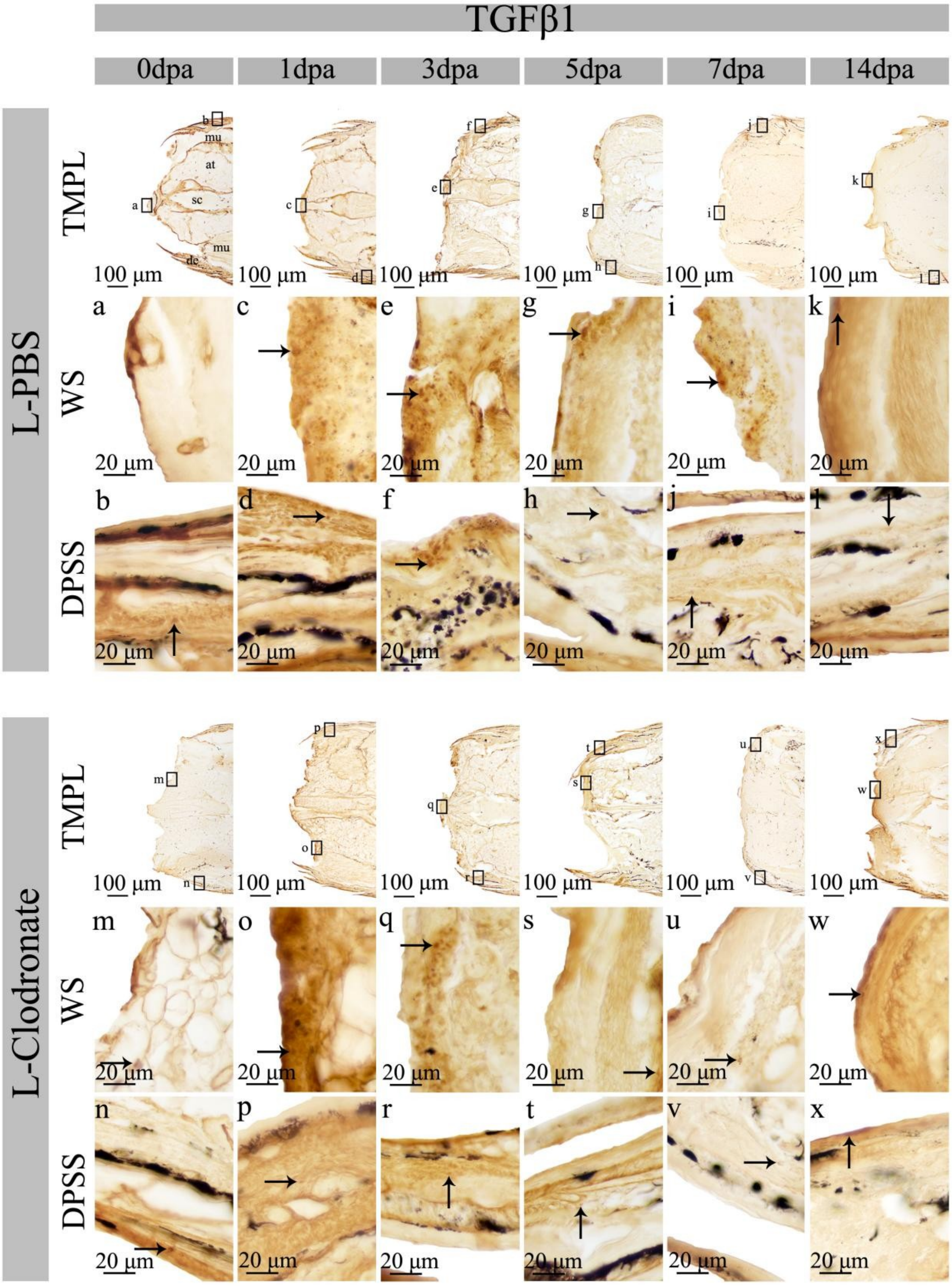

Supplementary Figure S7 Immunohistochemical localization of the pro-inflammatory factor TGFβ1 in the L-PBS group and L-Clodronate group of *S. tsinlingensis*

Note: Panels (a, c, e, g, i, and k) show the wound site under 40× magnification; Panels (b, d, f, h, j, and l) show the dermal layer of scales under 40× magnification. Arrows indicate TGFβ1-positive cells. at. Adipose tissue; de. Dermis; mu. Muscle; sc. Spinal cord; TMPL: The tissue morphology and positive labeling; WS:Wound site; DPS: The dermis of proximal scale to the stump surface, bar = 20 μm.

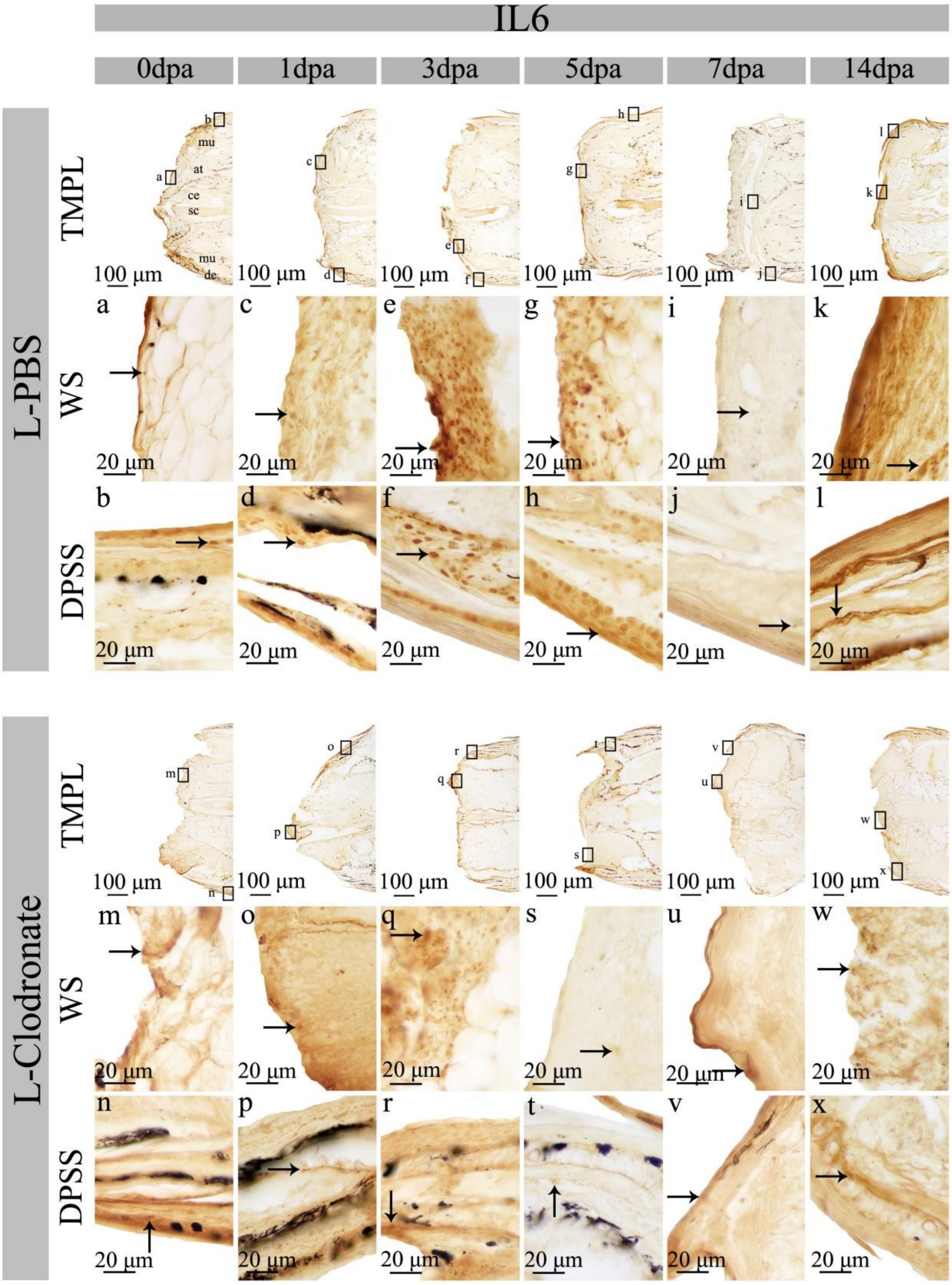

Supplementary Figure S8 Immunohistochemical localization of the anti-inflammatory factor IL6 in the L-PBS group and L-Clodronate group of *S. tsinlingensis*

Note: Panels (a, c, e, g, i, and k) show the wound site under 40× magnification; Panels (b, d, f, h, j, and l) show the dermal layer of scales under 40× magnification. Arrows indicate IL6-positive cells. at. Adipose tissue; de. Dermis; mu. Muscle; sc. Spinal cord; TMPL: The tissue morphology and positive labeling; WS:Wound site; DPS: The dermis of proximal scale to the stump surface, bar = 20 μm.

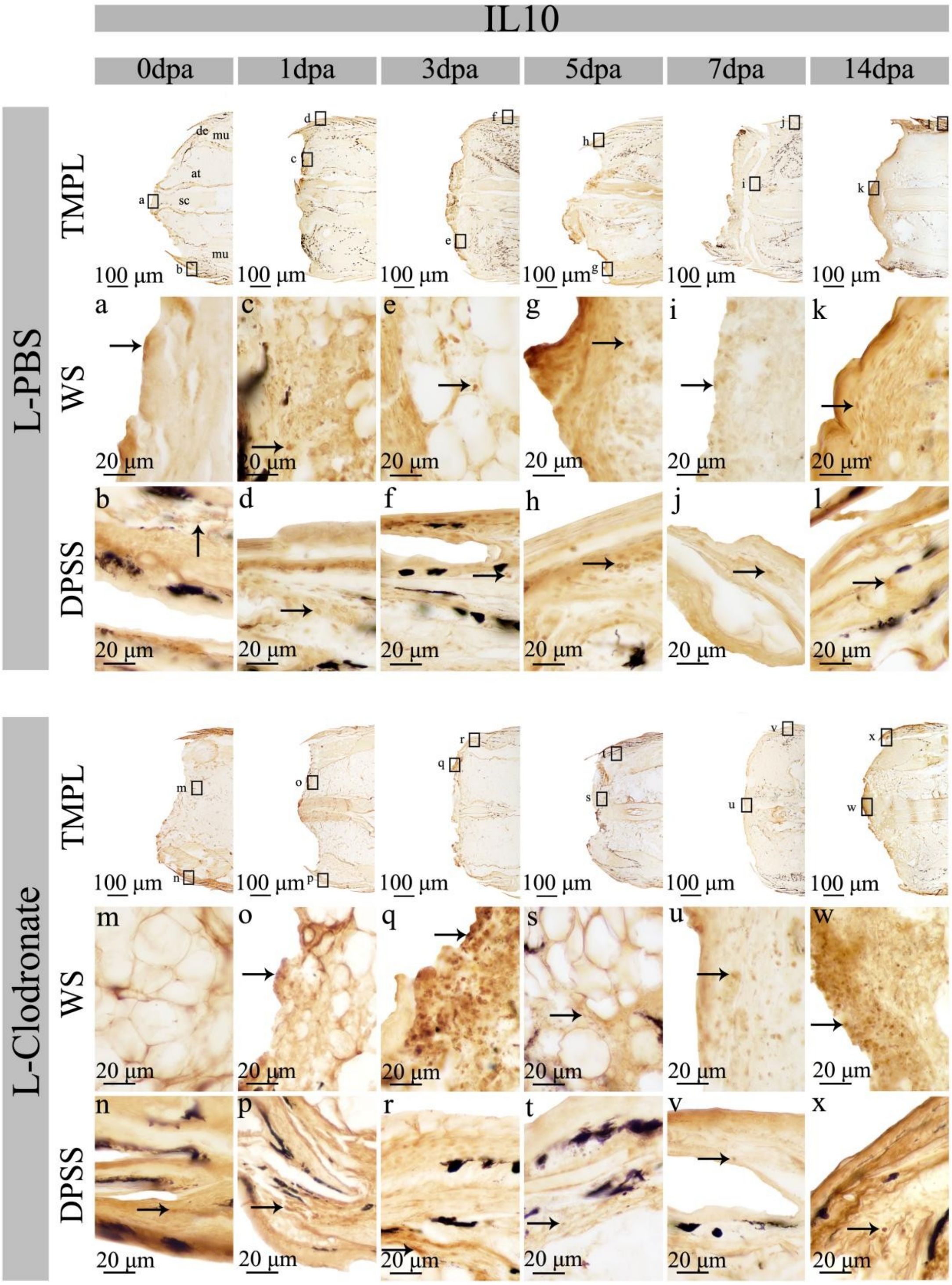

Supplementary Figure S9 Immunohistochemical localization of the anti-inflammatory factor IL10 in the L-PBS group and L-Clodronate group of *S. tsinlingensis*

Note: Panels (a, c, e, g, i, and k) show the wound site under 40× magnification; Panels (b, d, f, h, j, and l) show the dermal layer of scales under 40× magnification. Arrows indicate IL10-positive cells. at. Adipose tissue; de. Dermis; mu. Muscle; sc. Spinal cord; TMPL: The tissue morphology and positive labeling; WS:Wound site; DPS: The dermis of proximal scale to the stump surface, bar = 20 µm.

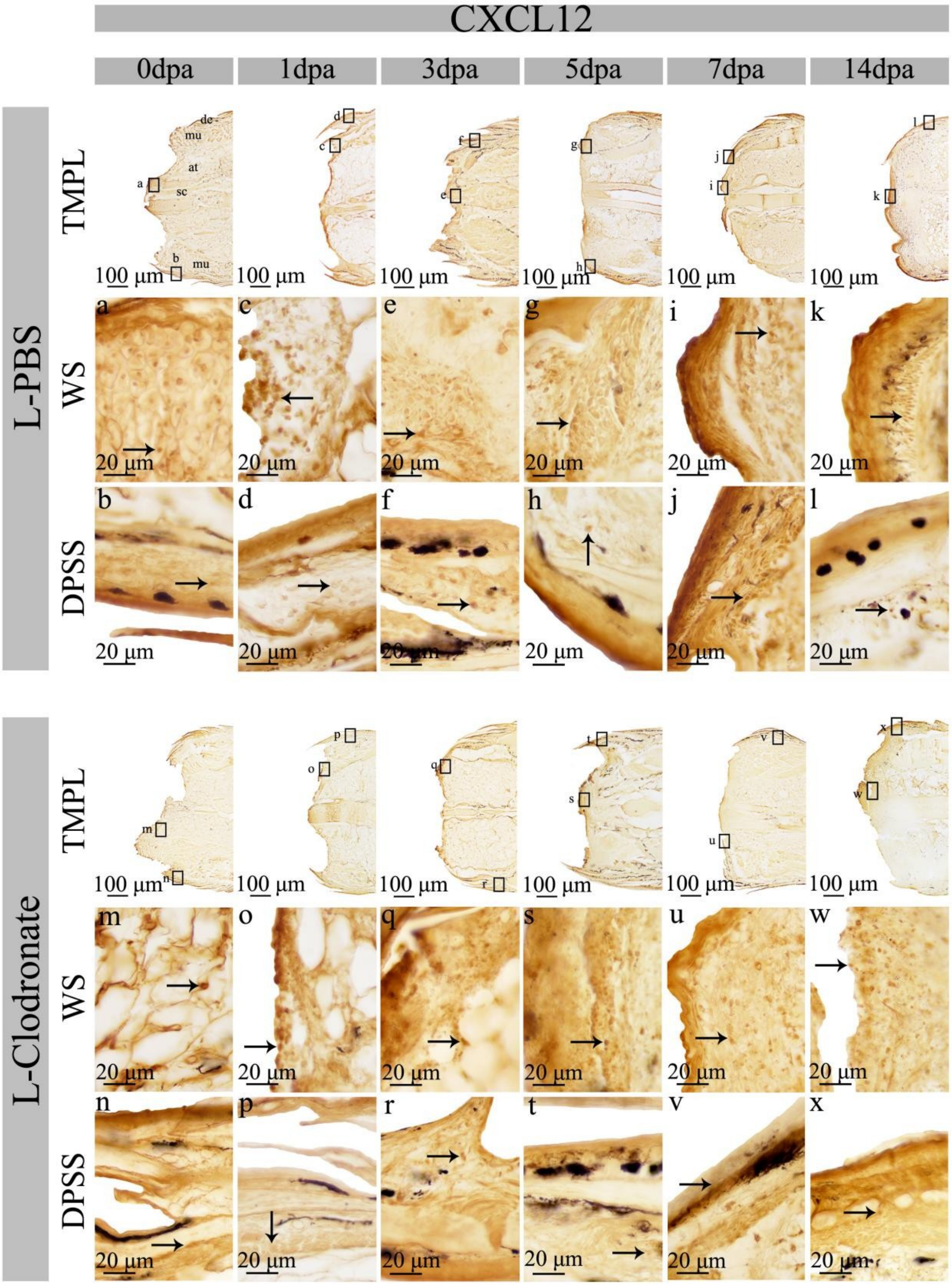

Supplementary Figure S10 Immunohistochemical localization of the chemokine CXCL12 in the L-PBS group and L-Clodronate group of *S. tsinlingensis*

Note: Panels (a, c, e, g, i, and k) show the wound site under 40× magnification; Panels (b, d, f, h, j, and l) show the dermal layer of scales under 40× magnification. Arrows indicate CXCL12-positive cells. at. Adipose tissue; de. Dermis; mu. Muscle; sc. Spinal cord; TMPL: The tissue morphology and positive labeling; WS:Wound site; DPS: The dermis of proximal scale to the stump surface, bar = 20 μm.

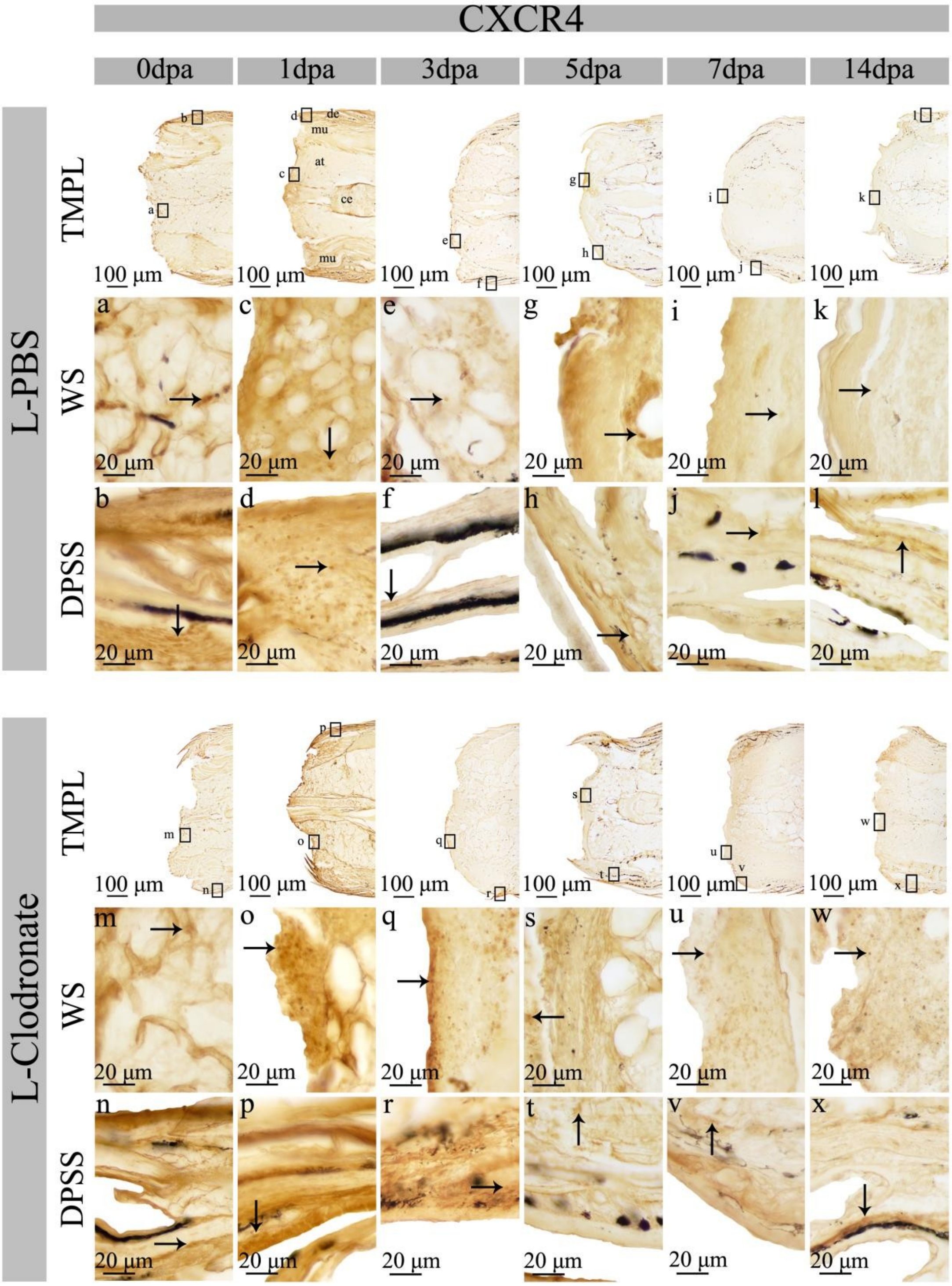

Supplementary Figure S11 Immunohistochemical localization of the chemokine CXCR4 in the L-PBS group and L-Clodronate group of *S. tsinlingensis*

Note: Panels (a, c, e, g, i, and k) show the wound site under 40× magnification; Panels (b, d, f, h, j, and l) show the dermal layer of scales under 40× magnification. Arrows indicate CXCR4-positive cells. at. Adipose tissue; de. Dermis; mu. Muscle; sc. Spinal cord; TMPL: The tissue morphology and positive labeling; WS:Wound site; DPS: The dermis of proximal scale to the stump surface, bar = 20 µm.

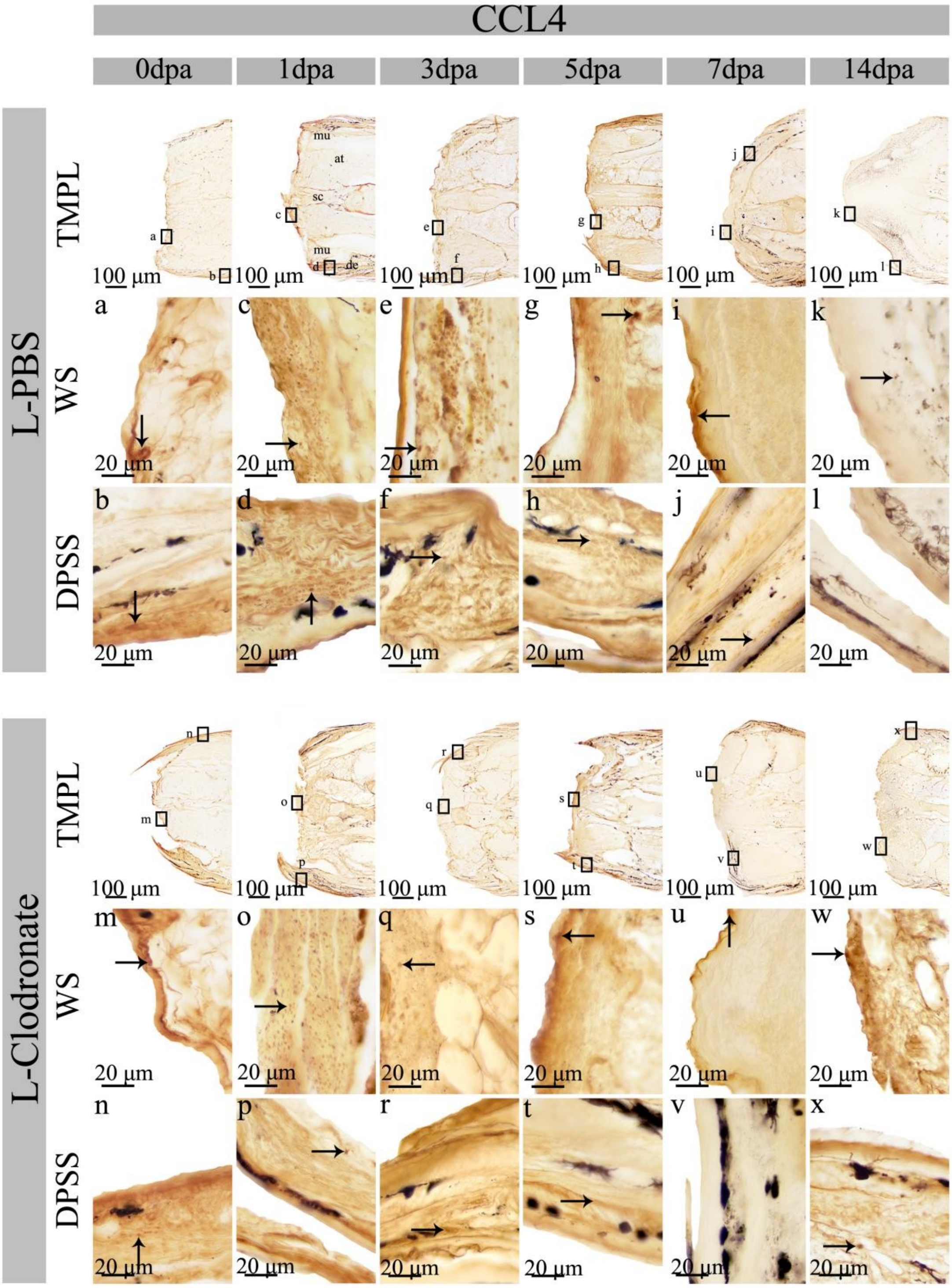

Supplementary Figure S12 Immunohistochemical localization of the chemokine CCL4 in the L-PBS group and L-Clodronate group of *S. tsinlingensis*

Note: Panels (a, c, e, g, i, and k) show the wound site under 40× magnification; Panels (b, d, f, h, j, and l) show the dermal layer of scales under 40× magnification. Arrows indicate CCL4-positive cells. at. Adipose tissue; de. Dermis; mu. Muscle; sc. Spinal cord; TMPL: The tissue morphology and positive labeling; WS:Wound site; DPS: The dermis of proximal scale to the stump surface, bar = 20 μm.

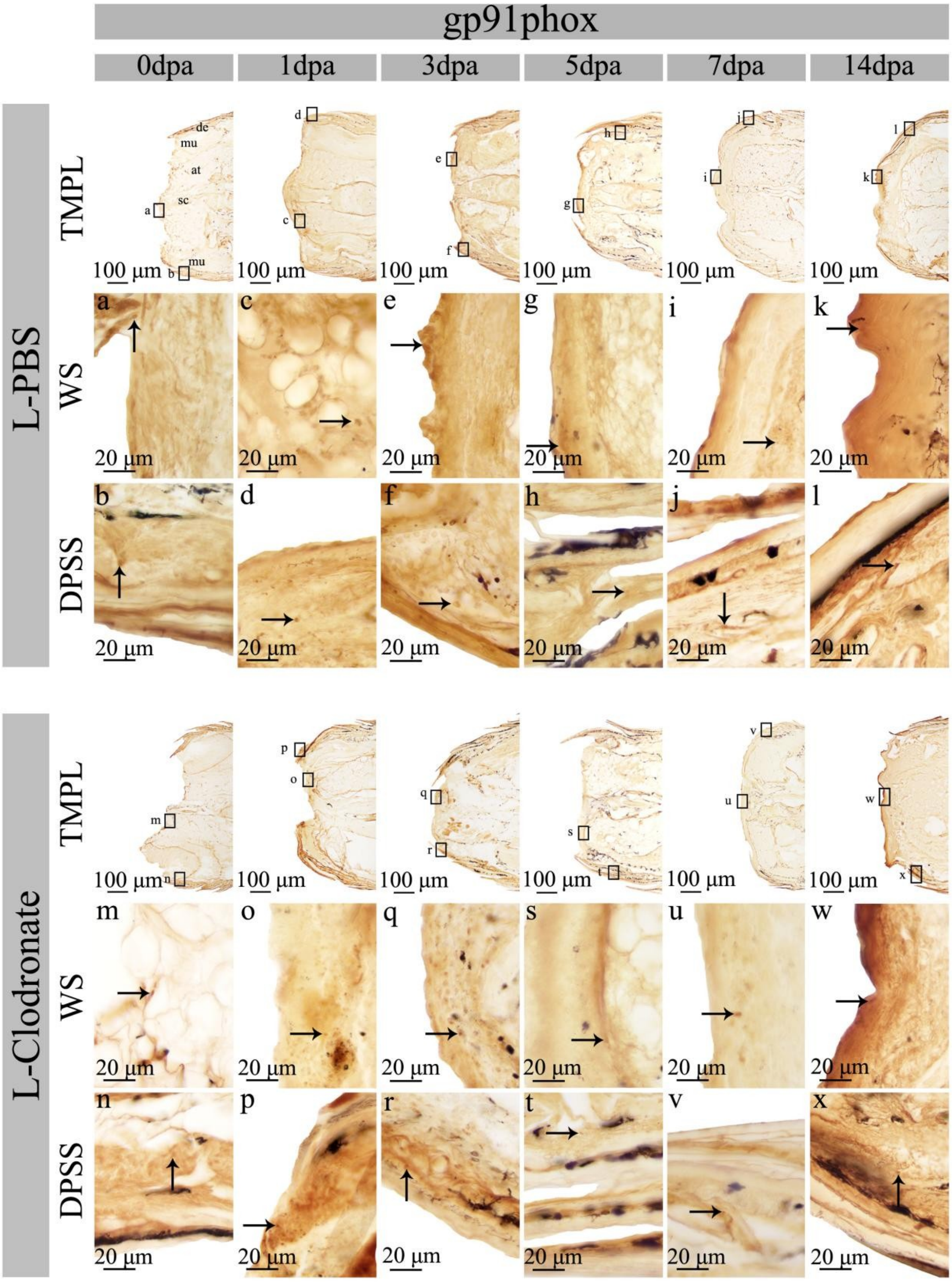

Supplementary Figure S13 Immunohistochemical localization of the oxidative stress factor gp91phox in the L-PBS group and L-Clodronate group of *S. tsinlingensis*

Note: Panels (a, c, e, g, i, and k) show the wound site under 40× magnification; Panels (b, d, f, h, j, and l) show the dermal layer of scales under 40× magnification. Arrows indicate gp91-positive cells. at. Adipose tissue; de. Dermis; mu. Muscle; sc. Spinal cord; TMPL: The tissue morphology and positive labeling; WS:Wound site; DPS: The dermis of proximal scale to the stump surface, bar = 20 μm.
